# Supplementary material for: Circular bioeconomy in African food systems: What is the status quo? Insights from Rwanda, DRC, and Ethiopia
Source: PLoS One. 2022 Oct 20;17(10):e0276319. doi: 10.1371/journal.pone.0276319 (PMC9584527; doi:10.1371/journal.pone.0276319)
Supplement: S1 Table — (DOCX) [file pone.0276319.s001.docx]

# S 1 Table. Regulatory initiatives or policies on CBE practices across countries

| Country | Policy / its initiative | Source | Ministry responsible | Guidance on CBE practices (what the policy promotes) |
| --- | --- | --- | --- | --- |
| Rwanda | Green Growth and Climate Resilience (GGCR) | RR, 2011 and expert interviews | Environment, and Kamonyi Municipality | - Use of clean energy like methane to replace oil-powered plants, and CO_2_ Forest sequestration - Resource recovery & reuse, agro ecology, organic composting, and wastewater irrigation, integrated soil management |
|  | Solid organic fertilizer specifications | RR, 2015 | Bureau of standards | - Developed standards for use of organic fertilizers - Prohibits use of raw human waste (urine & feces) citing impurity risks (specification of standards) |
|  | National Agriculture Policy | RR, 2017 | Agriculture | - Use of organic fertilizers to improve soil fertility - Research on bio-fertilizer technologies like composting |
|  | Strategic Plan for Transformation of Agriculture 2018-2024 | RR, 2017 | Agriculture | - Promote utilization of organic waste to improve and sustain soil fertility - Promote organic farm inputs e.g.vermi-composting, organic fertilizers, enriched compost) |
| DRC | Constitution of the DRC | DRC, 2011, and Expert interviews | Environment and Bukavu Municipality | - Management of waste (Articles 54, and 55) - Nature conservation |
|  | [Sur l’hygiene publique dans les agglomerations.](http://www.leganet.cd/Legislation/Droit%20Public/SANTE/O.74.345.28.06.1959.htm) | DRC, 1959; DRC, 2006; DRC 2015 and Expert interviews | Constitution | - General regulations on handling and processing waste to protect environment and enhance public health - General regulations about latrines, human waste (Articles 3,4, 6 and 10) |
|  | [Portant principes fondamentaux relatifs à la protection de l’environnent.](https://leganet.cd/Legislation/Droit%20economique/Agriculture/RDC%20-%20Loi%20agriculture%20principes%20fondamentaux-%2024%2012%202011.pdf) | DRC, 2011; DRC 2018 | Environment | - General regulations about agricultural inputs (Articles 28, and 31) |
| Ethiopia | Agricultural Sector Policy and Investment Framework (PIF) | FDRE, 2010 | Agriculture and Rural Development | - Use of organic fertilizers to manage soil fertility - Conservation of natural resources - Innovations that elevate soil organic carbon |
|  | Environmental Policy of Ethiopia | FDRE, 1997, and Expert Interviews | Environment, Forestry and Climate Change | - Use of organic matter, green manure, farmyard manures, and compost to improve soil structure - Use of agricultural residues, on-farm produced forage, and fodder as inputs for animal feeds |
|  | Environmental Pollution Control Proclamation (No. 300) | FDRE, 2002  FDRE, 2014 | Environment | - General guidelines about waste disposal |

**Bibliography to S 1 Table.**

*DRC (1959). 74-345 Sur l’hygiene publique dans les agglomerations. Accessed 3^rd^ September, 2019 from: http://www.leganet.cd/Legislation/Droit%20Public/SANTE/O.74.345.28.06.1959.htm*

*DRC (2006). Constitution de la Republique Democratique du Congo. Accessed 3^rd^ September, 2019 from: https://www.wipo.int/edocs/lexdocs/laws/fr/cd/cd001fr.pdf*

*DRC (2011). Portant principes fondamentaux relatifs à la protection de l’environnent, Kinshasa, DRC.*

*DRC (2015). N°15/026 relative à l’eau. Accessed on 5^th^ September, 2019 from: https://www.leganet.cd/Legislation/Droit%20economique/Eaux/Loi.15.026.31.12.2015.html#TIV*

*DRC (2018). N°18/035 fixant les principes fondamentaux à l’organisation de la santé publique. Accessed 19^th^ September 2019, from: http://www.leganet.cd/Legislation/Droit%20Public/SANTE/Loi.18.035.13.12.2018.html*

FDRE (2002). *Environmental Pollution Control Proclamation (No. 300).* Federal Democratic Republic of Ethiopia, Addis Ababa, Ethiopia

RR (2011). *Green Growth and Climate Resilience: National Strategy for Climate Change and Low Carbon Development.* Republic of Rwanda, Kigali, Rwanda
