# Supplementary material for: Circular bioeconomy in African food systems: What is the status quo? Insights from Rwanda, DRC, and Ethiopia
Source: PLoS One. 2022 Oct 20;17(10):e0276319. doi: 10.1371/journal.pone.0276319 (PMC9584527; doi:10.1371/journal.pone.0276319)
Supplement: S1 File — (PDF) [file pone.0276319.s002.pdf]

The RUNRES Generic Baseline Food Commodity Value Chain Questionnaire

BriefBackground and Justification

Kaplinsky (2004) defines a value chain as; "a full range of activities that are required to bring a product or service from conception, through the intermediary phases of production and delivery to final consumers, and final disposal after use". Value chains can therefore be for various final services/commodities, (ILO, 2015). A food value chain analysis enables proper understanding of specific food commodity value chains; unearthing the actors in these value chains, what they do, how they do it, where they are located, their relationships, and how these are sustained/governed. The food commodity value chain analysis also identifies opportunities along the value chain and guides actors to maximize on such opportunities, but as well identifies challenges or bottlenecks or gaps in the value chain, and helps inform appropriate interventions to correct these impediments, (WFP, 2010). WFP also adds that value chain analysis enables to know which actor in the chain earns how much, and what would be the optimal pricing of the food commodity. Although understanding the optimal prices of the food commodity could help chain actors redistribute benefits, but guidance on affordable prices by consumers would require the understanding of the consumers' incomes and expenditures, (WFP, 2010). Understanding consumers' incomes and expenditures can as well help predict consumers' Food Security statuses, (WFP, 2010). Therefore, alongside a comprehensive food commodity value chain analysis, an assessment of the consumers' incomes and expenditures is also necessary.

The RUNRES project seeks to engage stakeholders to map and quantify food systems following key food commodity value chains within city-regions of the selected four countries of the project's operations namely; Rwanda, the DRC, Ethiopia, and South Africa. Among the key food commodities of interest will be cassava in Rwanda, coffee in DRC, vegetables or maize in South Africa, and bananas or mangoes in Ethiopia. The mapping and quantification of these food systems will be aimed at understanding the current city-region food systems, and the challenges/bottleneck/gaps within these systems; and then with various stakeholders co-identify innovations/solutions/opportunities to alleviate these challenges. However, RUNRES is also interested in monitoring the effectiveness and the impact of the project activities within the mentioned city-regions. Therefore, RUNRES would need to implement a food commodity value chain tool below (for various actors), not only to understand the baseline status quo for later project impact assessment, but also to understand current food systems, their challenges, and possible innovations to alleviate these challenges.The consumers' incomes and expenditure, as well as the food security assessment questionnaire components would help complement the value chain tools with clear insights into the financial and food security status of the consumers, who must pay for all costs incurred in the value chain (Kaplinsky, 2004; WFP, 2010; and ILO, 2015).

According to the World Food Program (WFP), a value chain analysis (VCA) is built on a market system (a supply chain), detailing both structural and dynamic factors that affect contributions of each actor in the chain. These factors are very important all to be covered, and understood in detail in order to have a proper understanding of the value chain. ***Structural factors***of the VCA include:**1)**The characteristics of a food commodity – e.g. prices, quality, quantity (determined by end markets like buyers). **2)**The enabling environment – e.g. laws, regulations, policies, norms, infrastructure etc. i.e. factors facilitating or hindering the functioning of markets. **3)**Relationships (i.e. formal and informal linkages and information flows) between actors at different level of the VC. These relationships – particularly gender are critical in moving food commodities to end users (Tell who, where controls what). Where are the women, are all women dominated products and markets included? What are the power relations?**4)**Supporting markets along the value chain (e.g. financial services, telecommunications, irrigation, inputs delivery etc.). ***Dynamicfactors***(what keeps these actors together, what information is shared, how is their relationship evolving, where are their locations)–characterize how actors in the market system respond to opportunities and constraints (limitations).

In simplified food commodity value chain, actors include: input suppliers, farmers, middlemen (agents, assemblers/collectors, transporters), processors, wholesalers (importers/exporters), retailers, and final consumer. Therefore, in the RUNRES project we shall envision a Generic Baseline Food Commodity value chain as below:

Input Suppliers → Farmers/Producers → Middlemen (assemblers/collectors/transporters) → Processors → Wholesalers (Exporters/Importers) → [illegible]ailers → [illegible]s. This formation informs our actor specific questionnaire components.

General Guidelines and Instructions on Administering the Questionnaire

The tool is intended to serve RUNRES baseline purposes of establishing the status quo however, this baseline would also be used for the MEL activities, that would also entail empirical impact assessment. Therefore, we shall need to have statistically valid sample sizes, usually this is a minimum of 30 persons per group (actor segment) of analysis. This could do for the baseline. However, for the MEL goals, where we shall have to justify impact empirically, RUNRES shall need to use both statistically valid and representative sample sizes. Unfortunately, /fortunately, a number MEL activity would have to use the baseline (panel data scheme) hence, we must have the representative sample sizes from the start. Like Ben, had highlighted, the WFP (2010) recommends a sample size of minimum 400. However, some actor sections like input suppliers, middlemen, processors, wholesalers, and retailersmay not make this number. Also, certain key components of the MEL (quantitative impact assessment) are usually with the larger groups (actor sections) that is producers or consumers, for instance establishing changes in the food security status. Yet, other intermediate actors are also represented either as

consumers or producers. Therefore, we would stick to statistical validity (30 – 50) respondents for the input suppliers, middlemen, wholesalers, processors, and retailers where these are available. However, we may need representativeness in the farmers' section, moreover farmers can as well be consumers therefore and sometimes there is also a limited number of farmers / producers, hence we can aim at (150 – 200) respondents for farmers/producers. This is also based on the fact that certain empirical studies have been done with producers with case studies of between 100 – 300 respondents. Finally, it will be the consumer component where we would want as RUNRES to have highly both statistically valid, and scientifically population representative samples, especially when it would come to justifying our food security impacts as being representative in the city-regions of our presence. According to the oldest available empirical writing on representative population research samples, Krejcie and Morgan, (1970) recommend a sample size of 382 if the population size is 75,000, and a sample size of 384 if the population size is 1,000,000. The population of Arba Minch in 2017 was 200,373 persons, that of Kamonyi district was 386,531; that of Msunduzi municipality was 618,536 in 2011, and that of Bukavu was 870,954 in 2016. When I reached around for recent applications on determining the sample size, I came across the Raosoft software via: http://www.raosoft.com/samplesize.html , if we would aim at a 95% confidence interval for our results' predictive ability, and a 5% error margin, if I fill in the population for the respective city-regions, the recommended representative sample size comes to 384 persons; all in line with WFP (2010), and Krejcie and Morgan, (1970). This also tallies with regular formulae I have been used to using STATA. Therefore, at least for the consumers, if we want to claim representativeness for the city-regions for our food security improving initiatives, we may have to use a sample size of around 400. Moreover, as the MEL will go on in later years, some households may drop off or relocate, thus a reasonable target would be 450 – 500 consumers within each city-region. More importantly, this consumer segment, will be used for the empirical social aspects like establishing consumer preferences for possible RUNRES innovations, willingness to pay for these etc. and all these aspects shall have to be from representative samples for externally valid inferences; which is also a RUNRES goal, especially during scaling-up. Nevertheless, several arguments can be forwarded for smaller sample size, but apart from resources, we may not technically justify a lesser number. Moreover, for other actor segments we can argue that there were fewer numbers, but this cannot be with consumers. This sample would also allow us study the urban/rural dimensions more effectively – for instance we could target 200 urban consumers, 150 peri-urban consumers, and 150 rural consumers across the city-region.

Usually consumers are everywhere and exact respondents must be identified through a stratified random sampling procedure; starting with clear identification of the three strata within each city-region: Urban, Peri-Urban, and Rural. Then, villages/sectors/cells within each stratum must be listed, and a few selected randomly; then households within each randomly selected village/sector/cell must as well all be listed (this can be done with the help of the local administration to get the names of the persons within the village/cell/sector or if names can't be accessed randomly assigned numbers/figures representing each household (if total number of households is known) can be used and put in a common pull) and then respondent households are selected randomly. This procedure should as well work for producers. However, for other intermediate actors or even input sellers, the snowball methodology of identifying respondents must be used (if there is no central agency / office where these actors' details can be accessed from). For instance, I would start out with producers, and then ask them for reference to any of their input suppliers, then one input supplier would lead me to another and so on, until I would have the required number of respondents (40 – 50) (for possible MEL purposes as some may drop out, I bring the minimum to 40). I would still do this for middlemen identification, starting from farmers as the source; then processors starting from middlemen as the source, then wholesalers starting from processors as the source, and then retailers starting from wholesalers as the source. Each actor questionnaire must be administered independently (its own day, and own respondents) to limit enumerator mistakes.

Data should be collected with the help field enumerators – but with fulltime supervision by project coordinators, and frequently by the postdoc who must also make sure that the daily electronic data entry is done and correctly. If okayed, IITA would assist with an electronic template for data collection using ODK. The electronic version will ensure efficient monitoring, and timely data delivery. However, in case this is impossible, then a paper format may suffice. GPS readings will also be recorded for easier locational follow-up in later years (MEL aim). Moreover, GPS readings can also help give us exact mappings of targeted actors in the RUNRES city-regions. Because RUNRES is commodity focused, and the key commodities' value chains are all crops, livestock is silent here, however, livestock has been somewhat elaborated in the consumer incomes and expenditures component. Nevertheless, RUNRES innovations that will principally focus on waste recycling for animal feeds, will be aimed to bring out finer details on livestock contributions in the targeted city-region food systems.

Key References:

Coates, Jennifer, Anne Swindale, and Paula Bilinsky. 2007. Household Food Insecurity Access Scale (HFIAS) for Measurement of Household Food Access: Indicator Guide (V.3). Washington, D.C.: FANTA Project, Academy for Educational Development

FAO and FHI 360. 2016. Minimum Dietary Diversity for Women: A Guide for Measurement. Rome; FAO.

ILO, 2015. A Rough Guide to Value Chain Development: A Short Guide for Development Practitioners, Government and Private Sector Initiatives/ NadjaNutz and MertenSievers. International Labor Organization, Geneva.

Kaplinsky (2004).Spreading the Gains from Globalization: What can be learnt from Value Chain Analysis.*Problems of Economic Transition*. 47 (2): 74 – 115

Krejcie, R. V., & Morgan, D. W. (1970). Determining Sample size for Research Activities.*Educational and Psychological Measurement*. 30: 607 – 610

Swindale, Anne, and Paula Bilinsky. 2006. Household Dietary Diversity score (HDDS) FOR Measurement of Household Food Access: Indicator Guide (V.2), Washington, D.C.: FHI 360/FANTA

UBOS, 2017. Uganda National Household Survey 2016/17

WFP, 2010. How to Conduct a Food Commodity Value Chain Analysis

A. INPUT SUPPLIERS

Section 1: Enumeration details

1.	Country	
2.	District	
3.	Sector/Cell/Village	
4.	GPS reading of location	
5.	Name of enumerator	

Section 2: Household Bio data details

1.	Name of the respondent							
2.	Telephone contact of the respondent							
3.	Age of the respondent							
4.	Years of formal education of the respondent							
5.	Name, and distance to the nearest bigger town center							
6.	Gender of the respondent (*Tick right response*)	Male			Female			
7.	Marital status of the respondent	Married		Single		Divorced		Widow
8.	Number of persons under your household	Males			Females			

Section 3: Sector (Input) specific details (*Fertilizers/coffee seedlings/cassava cuttings etc.* ***TICK* *what matters)*

Select the actor along the food or waste value chains you are interviewing?

1.	What kind of farm inputs do you supply? *Tick what matters*	1 (Cassava cuttings) 2 (Coffee seedlings) 3 (Fertilizers) 4 (Tomato seeds) 5 (Banana suckers) 6 (Mango seedlings) 7 (Others – specify)
2.	Where do you get the inputs (fertilizers/cassava cuttings/coffee seedlings / tomato seedlings etc.) you supply to farmers? (*Tick what matters*)	1 (Government ministry/institution e.g. RAB/INERA etc.) 2 (non- government body/institution e.g. IITA, CIP, AGRA etc.) 3 (Own effort) 4 (Farmers' association/cooperative) 5 (Agro Dealers) 6 (Others – specify)
3.	How much quantity (Kgs/seedlings/cuttings) do you supply per week/month/season/year? (*Tick quantity units and timeline that apply*)	
4.	How much cost was incurredto supply the quantity in 3., with regards to each of the activities (a-f,)?*Record the exchange rate to USD*	a. Buying the raw material ______________ Francs<br>b. Processing the input __________________ Francs<br>c. Transportation and marketing ___________Francs<br>d. Licenses and other legal fees ____________Francs<br>e. Labor _____________________ Francs<br>f. All other costs ________________ Francs
5.	How much do you sell each unit (Kgs/seedlings/cuttings) of input supplied? (*if different quantities were sold at different unit prices, show the differences*)	______________________________Francs
6.	What is your opinion on the quality of the inputs you supply? (*Tick*)	1 (Bad) 2 (Fair) 3 (Good) 4 (Excellent) 5 (I don't know)
7.	Are there any laws/regulations/policies/norms guiding your input supplies business that you are aware about?	1 (Yes) 2 (No)
8.	If yes in 7., what are examples these that enable your business?	
9.	If yes in 7., what are examples these that hinder your business?	
10.	If no in 7, who then has power to regulate your business?	
11.	How many women/men are involved in this input (fertilizers/seedlings/cuttings) supply business in your sector/village?	Women ___________ Men __________
12.	What are the key roles of women/men in this business in your sector/village?	Women: 1 (financiers/owners) 2 (shop attendants) 3 (brokers) 4 (others – specify)<br>Men: 1 (financiers/owners) 2 (shop attendants) 3 (brokers) 4 (others – specify)
13.	How do you exchange information with your clients?	1 (person to person) 2 (Phone call) 3 (Phone SMS) 4 (Internet) 5 (Radio/TV) 6 (Other)
14.	What kind of information do you exchange?	1(new inputs arrivals) 2 (prices) 3 (performance of supplied inputs) 4 (Others – specify)
15.	What kind of people do you mainly target as clients?	1 (small scale farmers) 2 (large scale farmers) 3 Others (specify _________________)
16.	Which areas (locations/regions) are your clients located?	
17.	What keeps your clients committed to your inputs?	1 (Supplying genuine inputs) 2 (price cuts) 3 (all-time ready supplies) 4 (others ______)
18.	How about your suppliers, why do they commit to you?	
19.	How do you describe your relationship with clients?	
20.	Are there institutions willing to lend your business?	1 (Yes) 2 (No)
21.	If yes in 20., are these formal or informal financial institutions?	(Formal e.g. microfinances, Banks, etc.)2 (Informal e.g. farmer groups, family etc.)
22.	Are there any research/extension/government institutions providing you with new input technologies to sell to your clients?	1 (Yes) 2 (No)
23.	If yes in 22, what is the kind of these institutions?	1 (Government) 2 (Private) 3 (Non-for-Profit international) 4 (local groups) 5 (others)
24.	What is the major challenge/bottleneck/gap in supplying this input?	
25.	How can this in 24., challenge/bottleneck/gap be addressed?	

26.	What major opportunity do you see in the supply of this input?	
27.	How can you take up this opportunity in 26., effectively?	

B. FARMERS / PRODUCERS

Section 1: Enumeration details

1.	Country	
2.	District	
3.	Sector/Cell/Village	
4.	GPS reading of location	
5.	Name of enumerator	

Section 2: Household Bio data details

1.	Name of the respondent									
2.	Telephone contact of the respondent									
3.	Age of the respondent									
4.	Years of formal education of the respondent									
5.	Name, and distance to the nearest bigger town center									
6.	Gender of the respondent (*Tick right response*)	Male				Female				
7.	Marital status of the respondent	Married		Single		Divorced		Widow		
8.	Number of persons under your household	Males				Females				

Section 3: Sector (Farmers/Producers) specific details – Focus on the RUNRES Crop in the region

1.	What do you mostly produce on your farm? *Tick what matters*	1 (Cassava) 2 (Coffee) 3 (Tomato) 4 (Banana) 5 (Mango) 6 (Others – specify)
2.	Where do you get the inputs (fertilizers/cassava cuttings/coffee seedlings etc.) to use in your farm you? (*Tick what matters*)	1 (Government ministry/institution e.g. RAB/INERA etc.) 2 (non- government body/institution e.g. IITA, CIP, AGRA etc.) 3 (Own effort) 4 (Farmers' association/cooperative) 5 (Agro dealer) 6 (Others – specify)
3.	Where (locations/regions) are your input suppliers located?	
4.	What keeps your input suppliers committed to you?	1 (Prompt payment) 2 (long-time relationship) 3 (I pay if take credit) 4 (others _____)
5.	Where do you get fertilizers to use in your farm you?	1 (Government ministry/institution e.g. RAB/INERA etc.) 2 (non- government body/institution e.g. IITA, CIP, AGRA etc.) 3 (Own effort) 4 (Farmers' association/cooperative) 5 (Agro dealer) 6 (Others – specify) 7 (Never use fertilizers)
6.	Where (locations/regions) are your fertilizers suppliers located?	
7.	What keeps your fertilizers suppliers committed to you?	1 (Prompt payment) 2 (long-time relationship) 3 (I pay if take credit) 4 (others _____)
8.	How much quantity of cassava/coffee/mangoes/tomato/ banana (Kgs/baskets/sacks etc.) did you producelast season/year? (*Tick units and timeline that apply*)	____________ (Units ___) *if not Kgs please specify conversion factor (CF)*
9.	What was the cost of production incurred per last season/year? *Record the exchange rate to USD, timeline must be the same as in 3. Tick timeline that matters*	a. Buying the planting materials ______________ Francs<br>b. Renting land _________________ Francs<br>c. Paying labor __________________ Francs<br>d. If labor was not paid in c., how many man-days were used? _____ man days (1-man day = 6 hours)<br>e. Fertilizers ____________ Francs<br>f. Farm equipment purchase/repair ______ Francs<br>g. All other costs ________________ Francs
10.	What is your opinion on the quality of the planting materials that you used? (*Tick what matters*)	1 (Bad) 2 (Fair) 3 (Good) 4 (Excellent) 5 (I don't know)
11.	What is your opinion on the quality of the produce you harvested? (Tick)	1 (Bad) 2 (Fair) 3 (Good) 4 (Excellent) 5 (I don't know)
12.	Are there any laws/regulations/policies/norms guiding you on how production of this crop is done in your sector/village of operation?	1 (Yes) 2 (No)
13.	If yes in 11., what are these that enable you produce this crop better?	
14.	If yes in 11, what are these that hinder your better crop production?	
15.	If no in 11, who then has power to regulate your production of this crop?	
16.	How many women/men are involved in the production of this crop?	Women ___________ Men __________
17.	What are the key roles of women/men in the production of this crop?	Women: 1 (financiers/owners) 2 (Farming) 3 (product sellers) 4 (others – specify)<br>Men: 1 (financiers/owners) 2 (farming) 3 (product sellers) 4 (others – specify)
18.	Did you sell any of your farm produce for money?	1 (Yes) 2 (No) IF NO, skip to Question 25
19.	If yes in 15., how much quantity did you sell? And what was the unit price?	Quantity sold ________ Kgs/sacks/baskets etc. (*Specify and CF*)<br>Unit price _______ Francs/Birr/Rand (*Tick what matters*) (*if different quantities were sold at different unit prices, please show these differences*)
20.	In which form do you mostly sell your produce?	a) Flower stage b) Fresh harvest c) dried harvest with husks d) dried harvest without husks e) peeled fresh harvest f) peeled dry harvest g) dry pellets h) powder i) Others
21.	If not all produce was sold; how much quantity was consumed at home? Or was wasted during the process?	Quantity consumed ________ Kgs/sacks/baskets etc.<br>Quantity wasted ________ Kgs/sacks/baskets etc.
22.	Who (persons/agencies) do you sell too (clients)?	
23.	In what forms of the crop, is your final product to the client?	a) Flower stage b) Fresh harvest c) dried harvest with husks d) dried harvest without husks e) peeled fresh harvest f) peeled dry harvest g) dry pellets h) powder i) Others
24.	How do you exchange information with your clients?	1 (person to person) 2 (Phone call) 3 (Phone SMS) 4 (Internet) 5 (Radio/TV) 6 (Other)
25.	What kind of information do you exchange with clients?	1(available produce) 2 (prices) 3 (performance of old supplies) 4 (Others – specify)
26.	Which areas (locations/regions) are your clients located?	
27.	What keeps your clients committed to buying your produce?	1 (Supply genuine products) 2 (price cuts) 3 (all-time ready supplies) 4 (others ______)
28.	How do you describe your relationship with clients?	
29.	Are there institutions willing to lend you in the production of this crop?	1 (Yes) 2 (No)
30.	If yes in 29., are these formal or informal financial institutions?	(Formal e.g. microfinances, Banks etc.)2 (Informal e.g. farmer groups, family etc.)
31.	Are there any research/extension/government institutions providing you with new farm technologies (crops, equipment etc.) to use on your farm?	1 (Yes) 2 (No)
32.	If yes in 31, what is the kind of these institutions?	1 (Government) 2 (Private) 3 (Non-for-Profit international) 4 (local groups) 5 (others)
33.	What is the major challenge/bottleneck/gap in producing of this crop?	
34.	How can this in 33., challenge/bottleneck/gap be addressed?	

35.	What major opportunity do you see in the producing of this crop?	
36.	How can you take up this opportunity in 35., effectively?	

C. MIDDLEMEN (Assemblers/Collectors/Transporters/Fresh crop Traders)

Section 1: Enumeration details

1.	Country	
2.	District	
3.	Sector/Cell/Village	
4.	GPS reading of location	
5.	Name of enumerator	

Section 2: Household Bio data details

1.	Name of the respondent								
2.	Telephone contact of the respondent								
3.	Age of the respondent								
4.	Years of formal education of the respondent								
5.	Name, and distance to the nearest bigger town center								
6.	Gender of the respondent (*Tick right response*)	Male				Female			
7.	Marital status of the respondent	Married		Single		Divorced		Widow	
8.	Number of persons under your household	Males				Females			

Section 3: Sector (Middlemen) specific details – Focus on RUNRES Crop

1.	As a middleman (Assemblers/Collectors/Transporters/Traders) what crop do you deal in? *Tick what matters*	1 (Cassava) 2 (Coffee) 3 (Tomato) 4 (Banana) 5 (Mango) 6 (Others – specify)
2.	What do you exactly do with regards to this crop?	1 (Assemblers) 2 (Collectors) 3 (Transporters) 4 (Fresh crop Trader)
3.	Whosupplies you with the crop quantities you deal in? (*Tick what matters*)	1 (Farmers) 2 (Others – Specify)
4.	Which (areas/locations/regions) are your suppliers located?	
5.	What keeps your suppliers committed to you?	1 (Prompt payment) 2 (long-time relationship) 3 (I pay if take credit) 4 (others _____)
6.	How much quantity of this crop (Kgs/baskets/sacks etc.) did you deal in last season/year? (*Tick units and timeline that apply*)	____________ (Units ___) *if not Kgs please specify conversion factor (CF)*
7.	How much do you charge per unit of this crop, as you hand it to the next actor in the chain? (*if different quantities were sold at different unit prices, please show these differences*)	Francs
8.	What was the costs of dealing in this volume (in 6.,) of crop per last season/year? *Record the exchange rate to USD, timeline must be the same as in 3. Tick timeline that matters*	a. Vehicle hire / purchase ______________ Francs<br>b. Fuel _________________ Francs<br>c. Drivers labor __________________ Francs<br>d. If labor was not paid in c., how many man-days were used? _____ man days (1-man day = 6 hours)<br>e. Storage facilities' rent and maintenance ____________ Francs<br>f. Repair ______ Francs<br>g. Packaging _________________ Francs<br>h. Communication _______________ Francs<br>i. All other costs ________________ Francs
9.	What is your opinion on the quality of the crop product that you were handling as was from your supplier? (*Tick what matters*)	1 (Bad) 2 (Fair) 3 (Good) 4 (Excellent) 5 (I don't know)
10.	What is your opinion on the quality of the crop product, that you handled as you passed it on to the next actor in the chain? (Tick what matters)	1 (Bad) 2 (Fair) 3 (Good) 4 (Excellent) 5 (I don't know)
11.	Are there any laws/regulations/policies/norms guiding your business?	1 (Yes) 2 (No)
12.	If yes in 11., what are these that enable you deal this crop better?	
13.	If yes in 11., what are these that hinder your better dealing in this crop?	
14.	If no in 11, who then has power to regulate your dealings in this crop?	
15.	How many women/men are doing exact activities as yoursin this crop?	Women ___________ Men __________
16.	What are the key roles of women/men with regards to doing the same activities as you, around this crop?	Women: 1 (financiers/owners) 2 (store attendants) 3 (brokers) 4 (others – specify)<br>Men: 1 (financiers/owners) 2 (store attendants) 3 (brokers) 4 (others – specify)
17.	Which (persons/actors) do you sell/hand to after your activity (clients)?	
18.	In what form is your final product before you hand it to your client?	
19.	How do you exchange information with your clients?	1 (person to person) 2 (Phone call) 3 (Phone SMS) 4 (Internet) 5 (Radio/TV) 6 (Other)
20.	What kind of information do you exchange with clients?	1(new markets) 2 (prices) 3 (product management means) 4 (Others – specify)
21.	Which areas (locations/regions) are your clients located?	
22.	What keeps your clients committed to your services?	1 (Supply genuine products) 2 (price cuts) 3 (all-time ready supplies) 4 (others ______)
23.	How do you describe your relationship with clients?	
24.	Are there institutions willing to lend to you with regards to your activity?	1 (Yes) 2 (No)
25.	If yes in 24., are these formal or informal financial institutions?	(Formal e.g. microfinances, Banks, etc.) 2 (Informal e.g. farmer groups, family etc.)
26.	Are there any research/extension/government institutions providing you new technologies (communication, equipment etc.) to enhance business?	1 (Yes) 2 (No)
27.	If yes in 26, what is the kind of these institutions?	1 (Government) 2 (Private) 3 (Non-for-Profit international) 4 (local groups) 5 (others)
28.	What is the major challenge/bottleneck/gap in your business this crop?	
29.	How can this in 28., challenge/bottleneck/gap be addressed?	
30.	What major opportunity do you see in the dealing in this crop?	
31.	How can you take up this opportunity in 30., effectively?	

D. PROCESSORS

Section 1: Enumeration details

1. Country	
2. District	
3. Sector/Cell/Village	
4. GPS reading of location	
5. Name of enumerator	

Section 2: Household Bio data details

1. Name of the respondent							
2. Telephone contact of the respondent							
3. Age of the respondent							
4. Years of formal education of the respondent							
5. Name, and distance to the nearest bigger town center							
6. Gender of the respondent (*Tick right response*)	Male			Female			
7. Marital status of the respondent	Married		Single	Divorced		Widow	
8. Number of persons under your household	Males			Females			

Section 3: Sector (Processors) specific details – Focus on RUNRES Crop

1.	What crop do you process on your plan/station/site? *Tick what matters*	1(Cassava) 2 (Coffee) 3 (Tomato) 4 (Banana) 5 (Mango) 6 (Others – specify)
2.	Where do you get the raw material inputs for your processing? (*Tick what matters*)	1 (Farmers) 2 (Assemblers) 3 (collectors) 4 (Transporters) 5 (Fresh crop Traders) 6 (Others – specify)
3.	In what form are these raw materials when are supplied to you?	
4.	Where (locations/regions) are your raw materials suppliers located?	
5.	What keeps your raw materials suppliers committed to you?	1 (Prompt payment) 2 (long-time relationship) 3 (I pay if take credit) 4 (others _____)
6.	What exactly do you do to this raw materials when supplied to you?	
7.	How much quantity of this crop (Kgs/baskets/sacks etc.) did you process last season/year? (*Tick units and timeline that apply*)	____________ (Units ___) *if not Kgs please specify conversion factor (CF)*
8.	How much did you sell each unit of the final processed product? (*if different quantities were sold at different unit prices, please show these differences*)	
9.	What was the costs incurred in processing the above volume in 7.,last season/year? *Record the exchange rate to USD, timeline must be the same as in 3. Tick timeline that matters*	a. Building rent / constriction ______________ Francs<br>b. Machinery installation / repairs _________________ Francs<br>c. Electricity payments ____________ Francs<br>d. Water payments ___________________ Francs<br>e. Fuel for machinery ____________ Francs<br>f. Paying labor __________________ Francs<br>g. If labor was not paid in d., how many man-days were used? _____ man days (1-man day = 6 hours)<br>h. Packaging ________________ Francs<br>i. Transport _______________________ Francs<br>j. Marketing __________Francs<br>k. Processing inputs ________________ Francs<br>l. Administration ____________ Francs<br>m. Licenses and Taxes _________________ Francs<br>n. All other costs ________________ Francs
10.	What is your opinion on the quality of the crop raw materials that you received from your suppliers? (*Tick what matters*)	1 (Bad) 2 (Fair) 3 (Good) 4 (Excellent) 5 (I don't know)
11.	What is your opinion on the quality of the processed product that you produced? (Tick what matters)	1 (Bad) 2 (Fair) 3 (Good) 4 (Excellent) 5 (I don't know)
12.	Are there any laws/regulations/policies/norms guiding you on how to process this crop?	1 (Yes) 2 (No)
13.	If yes in 12., what are these that enable you process this crop better?	
14.	If yes in 12, what are these that hinder your better processing of this crop?	
15.	If no in 12, who then has power to regulate your production of this crop?	
16.	How many women/men are involved in the processing of this crop?	Women ___________ Men __________
17.	What are the key roles of women/men in processing this crop?	Women: 1 (finance/owners) 2 (store attendants) 3 (brokers) 4 (run machines) 5 (other<br>Men: 1 (finance/owners) 2 (store attendants) 3 (brokers) 4 (run machines) 5 (others)
18.	How much quantity of the processed product was not sold? And why?	Quantity processed and NOT sold ________ Kgs/sacks/baskets etc.<br>Reason ____________________________
19.	Who (persons/agencies) do you sell to (clients) your final product?	
20.	In what forms is your final product sold to the client?	
21.	How do you exchange information with your clients?	1 (person to person) 2 (Phone call) 3 (Phone SMS) 4 (Internet) 5 (Radio/TV) 6 (Other)
22.	What kind of information do you exchange with clients?	1(available market) 2 (prices) 3 (Product quality) 4 (Others – specify)
23.	Which areas (locations/regions) are your clients located?	
24.	What keeps your clients committed to buying your product?	1 (Supply genuine products) 2 (price cuts) 3 (all-time ready supplies) 4 (others ______)
25.	How do you describe your relationship with clients?	

26.	Are there institutions willing to lend you in the production of this crop?	1 (Yes) 2 (No)
27.	If yes in 26., are these formal or informal financial institutions?	(Formal e.g. microfinances, Banks, etc.) 2 (Informal e.g. farmer groups, family etc.)
28.	Are there any research/extension/government institutions providing you new technologies (communication, equipment etc.) to enhance business?	1 (Yes) 2 (No)
29.	If yes in 28, what is the kind of these institutions?	1 (Government) 2 (Private) 3 (Non-for-Profit international) 4 (local groups) 5 (others)
30.	What is the major challenge/bottleneck/gap in processing of this crop?	
31.	How can this in 30., challenge/bottleneck/gap be addressed?	
32.	What major opportunity do you see in the processing of this crop?	
33.	How can you take up this opportunity in 32., effectively?	
34.	What happens to the waste from your processing activities?<br>For what do you use the waste that you produce?<br>Into what do you process the waste ?<br>Do you collect waste from other persons for processing?<br>Do you pay them?<br>Do you process the waste traditionally or mechanically?<br>Are you the owner of equipment/facilities used to treat the waste?<br>Who is then the owner of the equipment/facilities you use to treat waste?<br>What kind of contract to you have with the equipment/facilities owner?<br>What do you do with the products you process from waste?<br>Are there challenges/bottlenecks/gaps in managing waste?<br>What is the major challenge/bottleneck/gap in managing the waste you generate?<br>How can the challenge/bottleneck/gap be addressed?<br>Do you see opportunities in waste you gerenate?<br>What major opportunity do you see from waste you generate?<br>How can you take up this opportunit, effectively?	Solid waste ___________________<br>Liquid waste ______________________

E. WHOLESALERS (Exporters/Importers)

Section 1: Enumeration details

1.	Country	
2.	District	
3.	Sector/Cell/Village	
4.	GPS reading of location	
5.	Name of enumerator	

Section 2: Household Bio data details

1.	Name of the respondent								
2.	Telephone contact of the respondent								
3.	Age of the respondent								
4.	Years of formal education of the respondent								
5.	Name, and distance to the nearest bigger town center								
6.	Gender of the respondent (*Tick right response*)	Male				Female			
7.	Marital status of the respondent	Married		Single		Divorced		Widow	
8.	Number of persons under your household	Males				Females			

Section 3: Sector (Wholesalers) specific details

1.	In what crop do you do you do your wholesale activities? *Tick what matters*	1(Cassava) 2 (Coffee) 3 (Tomato) 4 (Banana) 5 (Mango) 6 (Others – specify)
2.	Where do you get the raw material crops for your wholesale activities? (*Tick what matters*)	1 (Farmers) 2 (Assemblers) 3 (collectors) 4 (Transporters) 5 (Fresh crop Traders) 6 (Others – specify)
3.	In what form are these raw materials when are supplied to you?	
4.	Where (locations/regions) are your raw materials suppliers located?	
5.	What keeps your raw materials suppliers committed to you?	1 (Prompt payment) 2 (long-time relationship) 3 (I pay if take credit) 4 (others _____)
6.	What exactly do you do to in your wholesale activities?	1 (Exporter) 2 (Importer) 3 (Local wholesaler = sales locally)
7.	How much quantity of this crop (Kgs/baskets/sacks/Tons etc.) did you deal in (export or import or sale locally) last season/year? (*Tick units and timeline that apply*)	___________ (Units ___) *if not Kgs please specify conversion factor (CF)*
8.	How much did you sell each unit for? (*if different quantities were sold at different unit prices, please show these differences*)	Francs
9.	What was the costs incurred in handling the above volume in 7.,last season/year? *Record the exchange rate to USD, timeline must be the same as in 3. Tick timeline that matters*	a. Building rent / constriction _____________ Francs<br>b. Machinery installation / repairs ________________ Francs<br>c. Electricity payments ___________ Francs<br>d. Water payments _________________ Francs

	e. Fuel for machinery ____________ Francs<br>f. Paying labor __________________ Francs<br>g. Packaging ________________ Francs<br>h. Transport ________________________ Francs<br>i. Marketing __________Francs<br>j. Administration ____________ Francs<br>k. Licenses and Taxes _________________ Francs<br>l. All other costs _______________ Francs
10. What is your opinion on the quality of the crop raw materials that you received from your suppliers? (*Tick what matters*)	1 (Bad) 2 (Fair) 3 (Good) 4 (Excellent) 5 (I don't know)
11. What is your opinion on the quality of the product that you produced and sold to your clients? (Tick what matters)	1 (Bad) 2 (Fair) 3 (Good) 4 (Excellent) 5 (I don't know)
12. Are there any laws/regulations/policies/norms guiding your business?	1 (Yes) 2 (No)
13. If yes in 12., what are these that enable you do better in your business?	
14. If yes in 12, what are these that hinder you from doing better business?	
15. If no in 12, who then has power to regulate your business?	
16. How many women/men are involved in the wholesales of this crop?	Women ___________ Men __________
17. What are the key roles of women/men in wholesaling this crop?	Women: 1 (finance/owners) 2 (store attendants) 3 (brokers) 4 (run machines) 5 (other<br>Men: 1 (finance/owners) 2 (store attendants) 3 (brokers) 4 (run machines) 5 (others)
18. How much quantity of the product was not sold? And why?	Quantity NOT sold ________ Kgs/sacks/baskets etc.<br>Reason _____________________________
19. Who (persons/agencies) do you sell to (clients) your final product?	
20. In what forms is your final product sold to the client?	
21. How do you exchange information with your clients?	1 (person to person) 2 (Phone call) 3 (Phone SMS) 4 (Internet) 5 (Radio/TV) 6 (Other)
22. What kind of information do you exchange with clients?	1(Available market) 2 (prices) 3 (quality of supplied product) 4 (Others – specify)
23. Which areas (locations/regions) are your clients located?	
24. What keeps your clients committed to buying your product?	1 (Supply genuine products) 2 (price cuts) 3 (all-time ready supplies) 4 (others ______)
25. How do you describe your relationship with clients?	
26. Are there institutions willing to lend you in wholesaling of this crop?	1 (Yes) 2 (No)
27. If yes in 26., are these formal or informal financial institutions?	1 (Formal e.g. microfinances, Banks, etc.) 2 (Informal e.g. farmer groups, family etc.)
28. Are there any research/extension/government institutions providing you new technologies (communication, equipment etc.) to enhance business?	1 (Yes) 2 (No)
29. If yes in 28, what is the kind of these institutions?	1 (Government) 2 (Private) 3 (Non-for-Profit international) 4 (local groups) 5 (others)
30. What is the major challenge/bottleneck/gap in thiswholesale business?	
31. How can this challenge/bottleneck/gap in 30., be addressed?	
32. What major opportunity do you see in the wholesaling of this crop?	
33. How can you take up this opportunity in 32., effectively?	
34. What happens to the waste from your wholesaling activities?	Solid waste ______________________<br>Liquid waste __________________________

F. RETAILERS

Section 1: Enumeration details

1. Country	
2. District	
3. Sector/Cell/Village	
4. GPS reading of location	
5. Name of enumerator	

Section 2: Household Bio data details

1. Name of the respondent							
2. Telephone contact of the respondent							
3. Age of the respondent							
4. Years of formal education of the respondent							
5. Name, and distance to the nearest bigger town center							
6. Gender of the respondent (*Tick right response*)	Male			Female			
7. Marital status of the respondent	Married		Single		Divorced		Widow
8. Number of persons under your household	Males			Females			

Section 3: Sector (Retailers) specific details

1. In what crop do you do you retail? *Tick what matters*	1(Cassava) 2 (Coffee) 3 (Tomato) 4 (Banana) 5 (Mango) 6 (Others – specify)
2. Where do you get the raw material for retailing? (*Tick what matters*)	1 (Farmers) 2 (Assemblers) 3 (collectors) 4 (Transporters) 5 (Fresh crop Traders) 6 importers 7 (local wholesalers) 8 (Others – specify)
3. In what form are these raw materials when are supplied to you?	
4. Where (locations/regions) are your raw materials suppliers located?	
5. What keeps your raw materials suppliers committed to you?	1 (Prompt payment) 2 (long-time relationship) 3 (I pay if take credit) 4 (others _____)
6. How much quantity of this crop (Kgs/baskets/sacks/Tons etc.) did you retaillast season/year? (*Tick units and timeline that apply*)	____________ (Units ___ *if not Kgs please specify conversion factor (CF)*
7. How much did you sell each unit for? (*if different quantities were sold at different unit prices, please show these differences*)	Francs
8. What was the costs incurred in retailing the above volume in 6.,last season/year? *Record the exchange rate to USD, timeline must be the same as in 3. Tick timeline that matters*	a. Shoprent ______________ Francs<br>b. Storage facilities ______________ Francs<br>c. Electricity payments ____________ Francs<br>d. Paying labor __________________ Francs<br>e. Packaging ________________ Francs<br>f. Transport ________________________ Francs

	g. Marketing /Advertising __________Francs<br>h. Administration ____________ Francs<br>i. Licenses and Taxes _________________ Francs<br>j. All other costs ________________ Francs
9. What is your opinion on the quality of the stock that you received from your suppliers? (*Tick what matters*)	1 (Bad) 2 (Fair) 3 (Good) 4 (Excellent) 5 (I don't know)
10. What is your opinion on the quality of the product that you sold to your clients? (Tick what matters)	1 (Bad) 2 (Fair) 3 (Good) 4 (Excellent) 5 (I don't know)
11. Are there any laws/regulations/policies/norms guiding the retailing activities of this crop?	1 (Yes) 2 (No)
12. If yes in 11., what are these that enable you retail this crop better?	
13. If yes in 11, what are these that hinder your better retailing ofthis crop?	
14. If no in 11, who then has power to regulate your retailing of this crop?	
15. How many women/men are involved in retailing of this crop?	Women ___________ Men __________
16. What are the key roles of women/men in retailing this crop?	Women: 1 (finance/owners) 2 (store attendants) 3 (brokers) 4 (run machines) 5 (other<br>Men: 1 (finance/owners) 2 (store attendants) 3 (brokers) 4 (run machines) 5 (others)
17. How much quantity of the retail stock was not sold? And why?	Quantity NOT sold ________ Kgs/sacks/baskets etc.<br>Reason _____________________________
18. Who (persons/agencies) do you sell to (clients) your final product?	
19. In what forms is your final product sold to the client?	
20. How do you exchange information with your clients?	1 (person to person) 2 (Phone call) 3 (Phone SMS) 4 (Internet) 5 (Radio/TV) 6 (Other)
21. What kind of information do you exchange with clients?	1(Product forms) 2 (prices) 3 (new products supplies) 4 (Others – specify)
22. Which areas (locations/regions) are your clients located?	
23. What keeps your clients committed to buying your product?	1 (Supply genuine products) 2 (price cuts) 3 (all-time ready supplies) 4 (others ______)
24. How do you describe your relationship with clients?	
25. Are there institutions willing to lend you in retailing businessof this crop?	1 (Yes) 2 (No)
26. If yes in 25., are these formal or informal financial institutions?	1 (Formal e.g. microfinances, Banks, etc.) 2 (Informal e.g. farmer groups, family etc.)
27. Are there any research/extension/government institutions providing you new technologies (communication, equipment etc.) to enhance business?	1 (Yes) 2 (No)
28. If yes in 27, what is the kind of these institutions?	1 (Government) 2 (Private) 3 (Non-for-Profit international) 4 (local groups) 5 (others)
29. What is the major challenge/bottleneck/gap in the retailing of this crop?	
30. How can this in 29.,challenge/bottleneck/gap be addressed?	
31. What major opportunity do you see in the retailing of this crop?	
32. How can you take up this opportunity in 31., effectively?	
33. What happens to the waste from your retailing activities?	Solid waste ______________________<br>Liquid waste _________________________

G. CONSUMERS

Section 1: Enumeration details

1. Country	
2. District	
3. Sector/Cell/Village	
4. GPS reading of location	
5. Name of enumerator	

Section 2: Household Bio data details

1. Name of the respondent								
2. Telephone contact of the respondent								
3. Age of the respondent								
4. Years of formal education of the respondent								
5. Name, and distance to the nearest bigger town center								
6. Gender of the respondent (*Tick right response*)	Male				Female			
7. Marital status of the respondent	Married		Single		Divorced		Widow	
8. Number of persons under your household	Males				Females			

Section 3: Sector (Consumers' Incomes and Expenditures) specific details

**a. Household Incomes:**	
1. What is your major source of Household Income?	1 (From Agriculture activities) 2 (From non-agricultural activities)
2. How much do you earn per month from these non-agricultural activities?*This includes all incomes that contribute to the sustainability of the household*	a. Salaried employment ____________ Francs<br>b. Wholesale or Retail shop business ____________ Francs<br>c. Service provisions (transport/cleaning etc.) ___________ Francs<br>d. Brick laying and other crafts ____________ Francs<br>e. Remittances from friends and relatives __________ Francs<br>f. Dividends from businesses ___________ Francs<br>g. Pension where applicable _______________ Francs<br>h. Others (specify and total) ________________ Francs
3. How much do you earn per month/season from agricultural activities? (*Tick time of reference, and if season is ticked, tell how many months are in a season). If various items/units in a category were sold and at different rates (e.g. crops or livestock), list the different items on another paper or the back.*	a. Crops sales ____________ Francs (Qn'ty sold________ * unit Price _____)<br>b. Livestock sales __________ Francs (No. sold _______ * unit Price ______)<br>c. Sales of Livestock products ______ Francs (Qn'ty sold ____ * unit price _____)<br>d. Poultry sales ______________ Francs (No. sold _______* unit price ________)<br>e. Poultry products sales _________ Francs (Qn'ty sold ____ * unit price ________)<br>f. Apiculture (Honey) sales _________ Francs (Qn'ty sold ______ * unit price _______)<br>g. Fish sales _______________ Francs (Qn'ty sold ______ * unit price ________)

	h. Sale of forest/swamp products __________ (No. sold ______ * unit Price _______)<br>i. Provision of Agricultural labor ____________ Francs<br>j. Others (specify) ______________
**b. Expenditures: Household Non-food expenditure**	
4. How much per week/month/term/year do you spend on the following items? *Please specify the time dimension clearly. NOTE this includes all expenses on all household members*	a. House rent / repairs/ construction ____________ Francs _____ time unit<br>b. Medical insurance / bills/health ____________ Francs _____ time unit<br>c. Education for children/dependents __________ Francs _______ time unit<br>d. Transport fees/ licenses /fuel ____________ Francs _____________ time unit<br>e. Agricultural equipment purchases / repair __________ francs _______ time unit<br>f. Clothes purchases / repairs __________________ Francs ___________ time unit<br>g. Electricity bills ______________ Francs ___________ time unit<br>h. Water bills _________________ Francs _____________ time unit<br>i. Waste collection ______________ Francs ______________time unit<br>j. Cooking fuel (gas/firewood/charcoal/kerosene) ________ Francs _____ time unit<br>k. Security ___________ Francs ___________ time unit<br>l. Gifts (weddings/funerals etc.) _________ Francs __________ time unit<br>m. Business fees / licenses/ rent / taxes ___________ Francs ________ time unit<br>n. Communication (airtime/TV/Internet/web subscriptions ________ Francs ____ time<br>o. Home help / maids / support _____________ Francs ________ time unit<br>p. Furniture (chairs/beds/sofas etc.) ______________ Francs _______ time unit<br>q. Electronics purchases/repairs (fridge/lights/ pans etc.) ______ Francs ____ time units<br>r. Laundry (soap/paper/pads etc.) __________ Francs ______ time unit<br>s. Others (specify total) ______________ Francs _________ time unit

c. Household Food Expenditure (*Food item form specific quantities have been avoided for RUNRES aims (we don't aim at micronutrient consumption estimations. However, care must be taken to ensure that the respondent is allowed ample time to remember the total (expense/quantity) figures). Common products are stated independently.*

5. How much quantity of these food items or their products did you buy in the last 7 days and how much did you spend per unit of these foods?

Food item Consumed	Total ExpenseFr	Total Quantity	Units	Unit price	Form most consumed
1) Cassava					
2) Irish potato:					
3) Sweet potato (SP)					
4) Orange-fleshed SP					
5) Bananas					
6) Plantain					
7) Rice:					
8) Yam:					
9) Wheat:					
10) Bread:					
11) Maize:					
12) Yellow maize					
13) Sorghum:					
14) Sugarcane:					
15) Millet					
16) Milk:					
17) Poultry meat					
18) Animal meat					
19) Fish					
20) Eggs:					
21) Cabbages					
22) Onions					
23) Amaranth					
24) Spinach					
25) Chard					
26) Carrots					
27) Squashes / Pumpkins					
28) Other vegetables					
29) Mangoes					
30) Papayas					
31) Oranges					
32) Jack fruit					
33) Other fruits					
34) Lentils					
35) Beans					
36) Ground nuts					
37) Other nuts					
38) Peas					
39) Sim-sim					
40) Sugar					
41) Coffee					
42) Tea					
43) Salt					
44) Biscuits					
45) Chapati					
46) Doughnuts					
47) Mandazi					
48) Samusa					
49) Sodas					
50) Packed juices					

51)	Alcoholic drinks					
52)	Cigarettes/Tobacco					
53)	Cooking oil					
54)	Other drinks					
55)	Other foods					

Dark green leafy vegetables e.g. amaranth (red or green), spinach and chard.Vit- A rich vegetable/fruits e.g. Carrots, Squashes/pumpkins.Yellow maize.Mangoes.Papayas.

Section 4: Household Food Insecurity Access (HFIAS) Questions

	Question	Response options	Code
1.	In the past four weeks, did you worry that your household would not have enough food?	0 (No) (if no skip to Q2) 1 (Yes)	
1.a.	How often did this happen?	1 (Rarely (once or twice in the past four weeks)<br>2 (Sometimes (three to ten times in the past four weeks))<br>3 (Often (more than ten times in the past four weeks))	
2.	In the past four weeks, were you or any household member not able to eat the kinds of foods you preferred because of lack of resources?	0 (No) (if no skip to Q3) 1 (Yes)	
2.a.	How often did this happen?	1 (Rarely (once or twice in the past four weeks)<br>2 (Sometimes (three to ten times in the past four weeks))<br>3 (Often (more than ten times in the past four weeks))	
3.	In the past four weeks, did you or any household member have to eat a limited variety of foods due to lack of resources?	0 (No) (if no skip to Q4) 1 (Yes)	
3.a.	How often did this happen?	1 (Rarely (once or twice in the past four weeks)<br>2 (Sometimes (three to ten times in the past four weeks))<br>3 (Often (more than ten times in the past four weeks))	
4.	In the past four weeks, did you or any household member have to eat some foods that you really did not want to eat because of lack of resources to obtain other types of food?	0 (No) (if no skip to Q5) 1 (Yes)	
4.a.	How often did this happen?	1 (Rarely (once or twice in the past four weeks)<br>2 (Sometimes (three to ten times in the past four weeks))<br>3 (Often (more than ten times in the past four weeks))	
5.	In the past four weeks, did you or any household member have to eat a smaller meal than you felt you needed because there were not enough food?	0 (No) (if no skip to Q6) 1 (Yes)	
5.a.	How often did this happen?	1 (Rarely (once or twice in the past four weeks)<br>2 (Sometimes (three to ten times in the past four weeks))<br>3 (Often (more than ten times in the past four weeks))	
6.	In the past four weeks, did you or any other household member have to eat fewer meals in a day because there was not enough food?	0 (No) (if no skip to Q7) 1 (Yes)	
6.a.	How often did this happen?	1 (Rarely (once or twice in the past four weeks)<br>2 (Sometimes (three to ten times in the past four weeks))<br>3 (Often (more than ten times in the past four weeks))	
7.	In the past four weeks, was there ever no food to eat of any kind in your household because of lack of resources to get food?	0 (No) (if no skip to Q8) 1 (Yes)	
7.a.	How often did this happen?	1 (Rarely (once or twice in the past four weeks)<br>2 (Sometimes (three to ten times in the past four weeks))<br>3 (Often (more than ten times in the past four weeks))	
8.	In the past four weeks, did you or any household member go to sleep at night hungry because there was not enough food?	0 (No) (if no skip to Q9) 1 (Yes)	
8.a.	How often did this happen?	1 (Rarely (once or twice in the past four weeks)<br>2 (Sometimes (three to ten times in the past four weeks))<br>3 (Often (more than ten times in the past four weeks))	
9.	In the past four weeks, did you or any household member go a whole day and night without eating anything because there was not enough food?	0 (No) (if no questions are finished) 1 (Yes)	
9.a.	How often did this happen?	1 (Rarely (once or twice in the past four weeks)<br>2 (Sometimes (three to ten times in the past four weeks))<br>3 (Often (more than ten times in the past four weeks))	
