# Supplementary material for: Circular bioeconomy in African food systems: What is the status quo? Insights from Rwanda, DRC, and Ethiopia
Source: PLoS One. 2022 Oct 20;17(10):e0276319. doi: 10.1371/journal.pone.0276319 (PMC9584527; doi:10.1371/journal.pone.0276319)
Supplement: Attachment — Submitted filename: PONE-D-22-04453_reviewer_WM.pdf [file pone.0276319.s006.pdf]

PLOS ONE

Circular Bioeconomy in African Food Systems: What is the status quo? Insights from Rwanda, DRC, and Ethiopia.

--Manuscript Draft--

**Manuscript Number:**	PONE-D-22-04453
**Article Type:**	Research Article
**Full Title:**	Circular Bioeconomy in African Food Systems: What is the status quo? Insights from Rwanda, DRC, and Ethiopia.
**Short Title:**	Circular Bioeconomy in African Food System
**Corresponding Author:**	Haruna Sekabira, Ph.D.<br>International Institute of Tropical Agriculture<br>Kigali, RWANDA
**Keywords:**	Circular bioeconomy; awareness; acceptance; Food insecurity; Africa
**Abstract:**	Increasing global food insecurity amidst a growing population and diminishing production resources, renders the currently dominant linear production model insufficient to combat such challenges. Hence, a circular bioeconomy (CBE) model that ensures more conservative use of resources has become essential. Specifically, a CBE model that focuses on recycling and reusing organic waste is essential to close nutrient loops and establish more resilient rural-urban nexus food systems. However, the CBE status quo in many African food systems is not established. Moreover, scientific evidence on CBE in Africa is almost inexistent, thus limiting policy guidance to achieving circular food systems. Using a sample of about 2,100 farmers and consumers from key food value chains (cassava in Rwanda, coffee in DRC, and bananas in Ethiopia), we explored existing CBE practices; awareness, knowledge, and support for CBE practices; consumers' opinions on eating foods grown on processed organic waste (CBE fertilizers), and determinants of such opinions. We analysed data using generalized ordered logistic regressions in Stata. Results show that communities practice aspects of CBE, mainly composting, and are broadly aware, knowledgeable, supportive of CBE practices, and would broadly accept eating foods grown CBE fertilizers. Households with heads that used mobile phones, or whose heads were older, or married, or had a better education and agricultural incomes were more likely to strongly agree that they were knowledgeable and supportive of CBE practices and would eat CBE foods (foods grown on processed organic waste). However, the reverse was true for households that were severely food insecure or lived farther from towns. Rwandan and Ethiopian households compared to DRC were less likely to eat CB foods. Policies to stimulate CBE investments in all three countries were largely absent, and quality scientific evidence to guide their development and implementation is currently insufficient.
**Order of Authors:**	Haruna Sekabira, Ph.D.
	Elke Nijman
	Leonhard Späth, PhD
	Pius Krütli
	Marc Schut, PhD
	Bernard Vanlauwe, PhD
	Benjamin Wilde, PhD
	Kokou Kintche, PhD
	Speciose Kantengwa, MSc
	Abayneh Feyso, MSc
	Byamungu Kigangu, BSc
	Johan Six, PhD

Powered by Editorial Manager® and ProduXion Manager® from Aries Systems Corporation

Additional Information:	
**Question**	**Response**
**Financial Disclosure**<br><br>Enter a financial disclosure statement that describes the sources of funding for the work included in this submission. Review the submission guidelines for detailed requirements. View published research articles from *PLOS ONE* for specific examples.<br><br>This statement is required for submission and **will appear in the published article** if the submission is accepted. Please make sure it is accurate.<br><br>**Unfunded studies**<br>Enter: *The author(s) received no specific funding for this work.*<br><br>**Funded studies**<br>Enter a statement with the following details:<br>• Initials of the authors who received each award<br>• Grant numbers awarded to each author<br>• The full name of each funder<br>• URL of each funder website<br>• Did the sponsors or funders play any role in the study design, data collection and analysis, decision to publish, or preparation of the manuscript?<br>• **NO** - Include this sentence at the end of your statement: *The funders had no role in study design, data collection and analysis, decision to publish, or preparation of the manuscript.*<br>• **YES** - Specify the role(s) played.<br><br>* typeset	JS<br><br>grant no: 7F09521<br><br>Swiss Agency for Development and Cooperation (SDC)<br><br>https://www.eda.admin.ch/eda/en/fdfa/fdfa/organisation-fdfa/directorates-divisions/sdc.html<br><br>The funders had no role in study design, data collection and analysis, decision to publish, or preparation of the manuscript.
**Competing Interests**<br><br>Use the instructions below to enter a competing interest statement for this submission. On behalf of all authors, disclose any competing interests that could be perceived to bias this work—acknowledging all financial support and any other relevant financial or non-financial competing interests.	The authors have declared that no competing interests exist

Powered by Editorial Manager® and ProduXion Manager® from Aries Systems Corporation

This statement is required for submission and **will appear in the published article** if the submission is accepted. Please make sure it is accurate and that any funding sources listed in your Funding Information later in the submission form are also declared in your Financial Disclosure statement.<br><br>View published research articles from *PLOS ONE* for specific examples.<br><br>**NO authors have competing interests**<br><br>Enter: *The authors have declared that no competing interests exist.*<br><br>**Authors with competing interests**<br><br>Enter competing interest details beginning with this statement:<br><br>*I have read the journal's policy and the authors of this manuscript have the following competing interests: [insert competing interests here]*<br><br>* typeset	
**Ethics Statement**<br><br>Enter an ethics statement for this submission. This statement is required if the study involved:<br><br>• Human participants<br>• Human specimens or tissue<br>• Vertebrate animals or cephalopods<br>• Vertebrate embryos or tissues<br>• Field research<br><br>Write "N/A" if the submission does not require an ethics statement.<br><br>General guidance is provided below. Consult the submission guidelines for detailed instructions. **Make sure that all information entered here is included in the**	Our research did not contain any collection of plant, animal or other materials from nature. However, we only asked socio-economic questions from willing respondents. Nevertheless, we obtained an ethics approval permit in Rwanda, No.820/RNEC/2021

**Methods section of the manuscript.**<br><br>**Format for specific study types**<br><br>**Human Subject Research (involving human participants and/or tissue)**<br>• Give the name of the institutional review board or ethics committee that approved the study<br>• Include the approval number and/or a statement indicating approval of this research<br>• Indicate the form of consent obtained (written/oral) or the reason that consent was not obtained (e.g. the data were analyzed anonymously)<br><br>**Animal Research (involving vertebrate animals, embryos or tissues)**<br>• Provide the name of the Institutional Animal Care and Use Committee (IACUC) or other relevant ethics board that reviewed the study protocol, and indicate whether they approved this research or granted a formal waiver of ethical approval<br>• Include an approval number if one was obtained<br>• If the study involved *non-human primates*, add *additional details* about animal welfare and steps taken to ameliorate suffering<br>• If anesthesia, euthanasia, or any kind of animal sacrifice is part of the study, include briefly which substances and/or methods were applied<br><br>**Field Research**<br><br>Include the following details if this study involves the collection of plant, animal, or other materials from a natural setting:<br>• Field permit number<br>• Name of the institution or relevant body that granted permission	
**Data Availability**<br><br>Authors are required to make all data underlying the findings described fully available, without restriction, and from the time of publication. PLOS allows rare exceptions to address legal and ethical concerns. See the PLOS Data Policy and FAQ for detailed information.	No - some restrictions will apply

Powered by Editorial Manager® and ProduXion Manager® from Aries Systems Corporation

A Data Availability Statement describing where the data can be found is required at submission. Your answers to this question constitute the Data Availability Statement and **will be published in the article**, if accepted.<br><br>**Important:** Stating 'data available on request from the author' is not sufficient. If your data are only available upon request, select 'No' for the first question and explain your exceptional situation in the text box.<br><br>Do the authors confirm that all data underlying the findings described in their manuscript are fully available without restriction?	
**Describe where the data may be found in full sentences. If you are copying our sample text, replace any instances of XXX with the appropriate details.**<br><br>• If the data are **held or will be held in a public repository**, include URLs, accession numbers or DOIs. If this information will only be available after acceptance, indicate this by ticking the box below. For example: *All XXX files are available from the XXX database (accession number(s) XXX, XXX.*).<br>• If the data are all contained **within the manuscript and/or Supporting Information files**, enter the following: *All relevant data are within the manuscript and its Supporting Information files.*<br>• If neither of these applies but you are able to provide **details of access elsewhere**, with or without limitations, please do so. For example:<br><br>*Data cannot be shared publicly because of [XXX]. Data are available from the XXX Institutional Data Access / Ethics Committee (contact via XXX) for researchers who meet the criteria for access to confidential data.*<br><br>*The data underlying the results*	The data is available with the RUNRES project at IITA Rwanda partners, and the RUNRES online Polybox repository, and is fully accessible with authorization of the PI, Professor J. Six via johan.six@usys.ethz.ch.<br><br>The data is not yet publicly available because a period of 10 years is required since the inception of the funding (in 2019), before releasing the data to the public.

presented in the study are available from (include the name of the third party and contact information or URL).<br>• This text is appropriate if the data are owned by a third party and authors do not have permission to share the data.<br><br>* typeset	
Additional data availability information:	Tick here if the URLs/accession numbers/DOIs will be available only after acceptance of the manuscript for publication so that we can ensure their inclusion before publication.

Powered by Editorial Manager® and ProduXion Manager® from Aries Systems Corporation

Manuscript
Click here to access/download;Manuscript;CBE MANUSCRIPT PLOS ONE _REVISED.docx

Circular Bioeconomy in African Food Systems: What is the status quo? Insights from Rwanda, DRC, and Ethiopia.

Haruna Sekabira[1*], Elke Nijman[1], Leonhard Späth[2,3], Pius Krütli[3], Marc Schut[1,7], Bernard Vanlauwe [4], Benjamin Wilde [2], Kokou Kintche[5], Speciose Kantengwa[1], Abayneh Feyso[6], Byamungu Kigangu[5], Johan Six[2]

[1]Department of Natural Resources Management, International Institute of Tropical Agriculture, P.O. Box 1269 Kigali, Rwanda
[2]Department of Environmental Systems Science, Sustainable Agroecosystems, ETH Zurich, Universität strasse 2, 8092 Zurich Switzerland
[3]Department of Environmental Systems Science, Transdisciplinary Lab, ETH Zurich, Universität strasse 16, 8092 Zurich Switzerland
[4]Department of Natural Resources Management, International Institute of Tropical Agriculture, P.O. Box 30709-00100, Nairobi, Kenya
[5]Department of Natural Resources Management, International Institute of Tropical Agriculture, Km 18, Birava Bifurcation, Kalambo, DR Congo
[6]Department of Biology, College of Natural Sciences, Arba Minch University, P.O. Box 21. Arba Minch, Ethiopia

[7]Knowledge, Technology and Innovation Group of Wageningen University, Hollandseweg 1, 6706KN, Wageningen, the Netherlands

* Corresponding author: H.Sekabira@cgiar.org

Abstract

Increasing global food insecurity amidst a growing population and diminishing production resources, renders the currently dominant linear production model insufficient to combat such challenges. Hence, a circular bioeconomy (CBE) model that ensures more conservative use of resources has become essential. Specifically, a CBE model that focuses on recycling and reusing organic waste is essential to close nutrient loops and establish more resilient rural-urban nexus food systems. However, the CBE status quo in many African food systems is not established. Moreover, scientific evidence on CBE in Africa is almost inexistent, thus limiting policy guidance to achieving circular food systems. Using a sample of about 2,100 farmers and consumers from key food value chains (cassava in Rwanda, coffee in DRC, and bananas in Ethiopia), we explored existing CBE practices; awareness, knowledge, and support for CBE practices; consumers' opinions on eating foods grown on processed organic waste (CBE fertilizers), and determinants of such opinions. We analysed data using generalized ordered logistic regressions in Stata. Results show that communities practice aspects of CBE, mainly composting, and are broadly aware, knowledgeable, supportive of CBE practices, and would broadly accept eating foods grown CBE fertilizers. Households with heads that used mobile phones, or whose heads were older, or married, or had a better education and agricultural incomes were more likely to strongly agree that they were knowledgeable and supportive of CBE practices and would eat CBE foods (foods grown on processed organic waste). However, the reverse was true for households that were severely food insecure or lived farther from towns. Rwandan and Ethiopian households compared to DRC were less likely to eat CB foods. Policies to stimulate CBE investments in all three countries were largely absent, and quality scientific evidence to guide their development and implementation is currently insufficient.

Key words: Circular bioeconomy, awareness, acceptance, food insecurity, Africa

1. Introduction

The world is increasingly challenged to sustainably feed its ever increasing population amidst diminishing resources and worsening climate change impacts (van Ittersum et al. 2016; Recous et al. 2019). Therefore, without sustainable production and consumption systems, achievement of several of the strategic sustainable development goals (SDGs), like poverty alleviation, food security, environmental health, and sustainable cities is in jeopardy. (FAO, 2018; Kershaw et al. 2021; Muscat et al. 2021). Moreover, part of the causes for the worsening global food production and consumption systems' situation is humans, whose dignified existence is the prioritized aim of the SDGs. For instance, the linear model of resource use employed by modern food production and consumption systems is blamed for depletion of resources (Majumdar et al. 2016; Kershaw et al. 2021). Under this linear model, (Geissdoerfer et al. 2017; Korhonen et al. 2017; 2018; Kirchherr et al. 2018; EMF, 2019), production of food occurs in rural areas, while most consumption occurs in urban areas, where most waste (food and human) accumulates in uncontrolled dumpsites, rudimentary sanitation facilities, or is released into the environment (Kaza et al. 2018; RUNRES 2019; Muscat et al. 2021). However, organic waste contains valuable soil nutrients, and not reusing them in food systems where they were mined breaks nutrient loops, which results in long term soil nutrient depletion (van der Wiel et al. 2019). Indeed, the globe is ultimately circular in use of her resources, however, if resources are mined heavily and the waste or biproducts from these resources are not reused in the particular food system, then the resource use model is a linear one, (Geissdoerfer et al. 2017; Kershaw et al. 2021) Such a linear production system renders the rural-urban food system nexus non-resilient. Moreover, accumulation of waste in urban areas constitutes an environmental concern that threatens both human and environmental health (Ferronato & Toretta, 2019). In essence, the linear model of modern food production is vulnerable to climate change effects, and contributes to food insecurity, poverty, poor human health, soil degradation, and biodiversity loss – hence it is unsustainable, (Kaza et al. 2018; Kershaw et al. 2021; Muscat et al. 2021).

On that basis, global efforts have recently focused on shifting from a linear model of resource use to a circular bioeconomy (CBE) one. A CBE model reduces waste produced across a given supply chain, utilizes food waste and agricultural by-products, and recycles nutrients (Jurgilevich et al. 2016; Carus and Dammer, 2018; Kershaw et al. 2021; Muscat et al. 2021). Circular bioeconomy is a combination of circular economy and bioeconomy, (Kershaw et al.

2021). A circular economy aims to rearrange the linear take–make–use–dispose resource use model in production and consumption systems with a circular formation that recycles and reuses resources back into the particular system, while limiting negative externalities, (Geissdoerfer et al. 2017). On the other hand, bioeconomy generally aims to provide good and services sustainably through production and use of biological resources, processes, and products, that ultimately replace fossil resources, (Kershaw et al. 2021; Muscat et al. 2021). Therefore, under the CBE food system, production would still mostly occur in rural areas, and consumption in town centres. However, after urban consumption, organic waste (food, household, and human) would be collected, recycled, and returned to rural areas for reuse on farms (EMF, 2019; Williams, 2019). Because caution is undertaken, under the CBE model to protect and restore the healthiness of production ecosystems through evading non-essential products and waste from production of essential products, while prioritizing efficient energy use and biomass streams for basic human needs; the CBE model in its entirety differs from the usual mere recycling of waste, (Muscat et al. 2021). In fact, Carus and Dammer (2018), assert that a CBE model aims to provide renewable biological resources and converts such resources and their waste streams into products of added value, for instance food, animal feed, bioenergy, or other bio-based products while leading to more efficient, and sustainable agricultural practices, while creating new opportunities for investment, business, and jobs. Therefore, reusing biologically recycled biomass and other waste in areas of a particular food system, where production occurs aiming at creating more efficient, restorative, and sustainable food systems, is what makes such a food system a CBE one, (Muscat et al. 2021). Reusing recycled waste in this manner would close nutrient loops, replenish soil nutrients and soil organic matter, all of which would increase sustainable farm productivity (van der Wiel et al. 2019). More so in African food systems that are characterized by low farm inputs, there is potential to transform organic waste into useful farm inputs (Frankema, 2014). Increased crop or animal productivity from use of recycled nutrients[1] could also increase household incomes via improved crop and animal yields or reduce household expenditure via low food costs caused by increased food supply. Thus, poverty as well as other household welfare challenges (education, health, nutrition, housing, communication etc.) could be addressed via this income pathway. The CBE

[1] Assuming low production costs of efficiently processed inputs (recycling done in homes or at dumpsites to avoid transporting bulky municipal organic waste expensively from dumpsites to homes, or original waste is sorted at source to minimize additional costs in sorting), since organic waste is abundantly available

model would also ensure that waste does not become an environmental problem in towns, where it harbours pathogens that threaten human health, emits greenhouse gases (GHG), or pollutes air, and thus lower the environmental quality necessary for proper human health (Recous et al. 2019; EMF, 2019). The use of organic compost from recycled waste could also reduce the need for inorganic fertilizers, thus reducing costs on buying fertilizers (Liverpool-Tasie et al. 2016).

However, most farmers and consumers in Africa are familiar with the linear model (produce food, and take it to consumption areas like towns and leave the waste to be disposed of there without being reused where food was produced), and view organic waste as valueless,(Bello & Ismail, 2016; Recous et al. 2019). Furthermore, in some communities, even in rural areas, it is perceived as against social and cultural norms to harvest waste as a resource, especially human waste (Chunga et al. 2016). Nevertheless, a CBE model must have an approach backed by local policies, that is compatible with key social, cultural, ethical, and ecological frameworks in order to achieve a sustainable pathway, (Kershaw et al. 2021; Issa et al. 2019) Sadly, policies in most African countries do not prioritize organic waste recycling and reuse, and in some countries, CBE policies are largely inexistent (Kaza et al. 2018; Gatune et al. 2021). Further problematic is that scientific evidence is currently insufficient to guide global food systems' policies on addressing gaps around CBE (Jurgilevich et al. 2016; Geissdoerfer et al. 2017; Gatune et al. 2021). Despite good CBE prospects, actors in food systems need to understand the potential of CBE concepts, and how these changes can be effectively implemented (van Ittersum et al. 2016). With our study, we contribute to enabling food systems' actors in studied African city-regions to understand the local communities' status quo on CBE practices vis-à-vis community attitudes, perceptions, and preferences. Moreover, Geissdoerfer et al. (2017) note that, to date, the principle focus of CBE research has been on developed countries dominated by (in decreasing importance[2]): China, UK, Netherlands, USA, Italy, Japan, Sweden, Germany, Belgium (up to 25 western countries). Africa was not featured in recent developments of CBE, perhaps because severe consequences of the linear model have been most pronounced in highly industrialized countries.

However, the rapidly increasing African urban population is as well exposed to the severe consequences of food insecurity and poor sanitation in urban centres due to waste accumulation and excessive soil nutrients depletion in rural areas (Kaza et al. 2018; RUNRES, 2019). If this

[2] China being the most researched

non-restorative use of resources is not reversed, sustainable development in Africa grounded on food production and consumption systems could be difficult to attain (Kirchherr et al. 2017; 2018; Kaza et al. 2018; Muscat et al. 2021; Gatune et al. 2021). Therefore, we contribute towards sustainable food systems by analysing CBE practices in four African city regions based on data from 2,175 households. This data has been collected by the project "Rural-Urban Nexus: Establishing a Nutrient Loop to Improve City Region Food Systems Resilience (RUNRES; https://runres.ethz.ch/)", funded by the Swiss Development Agency (SDC). RUNRES aims to establish a circular bioeconomy in 4 African city regions by using cost-effective, and socially acceptable CBE innovations to close nutrient loops through valorisation of organic waste in food systems. RUNRES operates in four African city regions, focusing on four food commodity value chains: cassava in Kamonyi (Rwanda), coffee in Bukavu (Democratic Republic of Congo (DRC)), bananas in Arba Minch (Ethiopia), and vegetables in Msunduzi (South Africa). We used data from all city regions, except Msunduzi, South Africa (due to data unavailability) to investigate CBE status quo in these city-regions. Specifically, we sought answers to the following questions:

1) What CBE practices do currently exist in the respective city-regions' food systems?
2) Are communities currently aware, knowledgeable, or supportive of CBE practices?
    a. What factors do explain such CBE awareness, knowledge, and support?
3) What are communities' opinions on consuming foods grown on CBE fertilizers?
    a. What factors do explain these consumption opinions?
4) Are there policies guiding CBE practices in the selected city-regions?
    a. If yes; what are these policies' guidance on CBE practices?

Addressing research questions above generated knowledge on where we are with CBE practices in the mentioned city-regions. This knowledge would aggregately guide efforts on successfully designing and scaling economically suitable, socially, and legally acceptable CBE innovations. Economic suitability, social and legal acceptability are attributes that render CBE innovations desirable and sustainable (Kaza et al. 2018; Carus and Dammer, 2018; RUNRES, 2019; Muscat et al. 2021; Kershaw et al. 2021).

The rest of the paper is presented as follows: next we present the methods and data used for this study, after which we present results and then discuss them separately. Finally, we conclude with concise guidance on policy areas. time

2. Methods and Data

2.1. The Circular Bioeconomy Concept

For this study, we adopted the circular economy (CE) definition of Kirchherr et al. (2017), which broadly defines the CE concept as "a system that replaces the 'end-of-life' notion, through a combination of principles and practices that not only reduce waste, but recycle and reuse available waste. However, literature also takes note of multiple and somewhat divergent interpretations of the CE concept (Kirchherr et al. 2017; Korhonen et al. 2018). In fact, Kirchherr et al. (2017) adds that some authors confuse the CE concept with just recycling, while he argues that the CE concept must include design elements that reduce waste, recycle it, and reuse it efficiently. However, CE has been linked with bioeconomy which broadly refers to the use of biological resources, processes, and products to sustainably produce consumables, (Kershaw et al. 2021). Therefore, linking the two, a CE focusing on the reduction and reuse of organic waste from food supply chains has been called a circular bioeconomy (CBE) (Carus and Dammer, 2018; Kershaw et al. 2021). More specifically, El-Chichakli et al. (2016) expounded on this by making it clearer that a circular bioeconomy is a model that recycles and reuses organic waste through biological processes like fermentation, biotechnology, and molecular biology. Not reusing organic and waste products in a food system, where production takes place renders a system linear and breaks the nutrients cycle because decomposing waste for instance in towns doesn't avail these nutrients to be reused in food production in rural areas (Muscat et al. 2021). Therefore a CBE model must ensure an efficient cycle that biologically recycles and reuses products and waste resources after consumption back in food production to render the system circular, (Carus and Dammer, 2018). Given the focus of RUNRES on the capture, biological processing, and reuse of available organic waste in waste streams in the four-city regions, we focus on CBE in this study**.**

2.2. Research Area, Commodities, Sampling, and Data

We analysed three food commodity value chains, and their systems, in three different African countries where RUNRES is promoting CBE based innovations[3]. RUNRES city-regions were purposively selected because of their dominance in growing the food commodities of interest,

[3] Data analyzed here were collected before RUNRES started implementing CBE innovations to properly understand CBE status quo

and their existing connections with project partners and stakeholders. The main project private sector partners in all countries were garbage collection companies, while main public partners were municipal authorities. Once city-regions had been selected, we identified the most relevant food commodities within each city-region. Through this preliminary and participatory work, our stakeholder networks selected cassava in Kamonyi, Rwanda, coffee in Bukavu, DRC, and bananas in Arba Minch, Ethiopia (RUNRES, 2019). Within each city region, we selected various administrative sub-divisions randomly (we listed all sub-divisions from each city-region, and randomly selected those we worked with). We then selected respondent households through a systematic random sampling of available household lists from those respective sub-divisions. In Rwanda, we obtained household lists from sector (sub-division) offices for farmers and consumers. For farmers, and consumers, whose numbers would be generated sufficiently, we chose a household at every fifth interval in Rwanda, sixth interval in Ethiopia, and seventh interval in DRC to be surveyed. These intervals were used to avoid a sample dominated by certain villages/cells (smallest administrative unit) within the city-region. In Ethiopia, household lists were obtained from *Kabele* (sub-division) offices for both farmers and consumers. While in DRC, a farmers' list was obtained from the national coffee office in Bukavu and validated by coffee cooperatives. A consumers' list was obtained from Bukavu municipality offices and validated by sanitation service providers. Other value chain actors like inputs sellers, middlemen, processors, wholesalers, and retailers, were identified in a snowball sampling methodology beginning with farmers. This was because these actors were very few and were sparsely distributed. Fewer numbers could not be used to draw meaningful statistical inferences across all countries; hence, we do not use these other actors in our empirical analysis. Nevertheless, we use some of the observations and lessons learned from these actors, to present and discuss empirical results in this study, and draw plausible recommendations. We present all interviewed value chain actors in Table 1.

Following WFP (2010) protocols for representative samples in food commodity value chain analysis, we set 400 households per value chain actor segment as our target sample size. However, from field explorations, we found that some actors' segments, were either non-existent or had far much less than 400 households. Numbers dwindled more when we executed systematic random sampling for participants. However, because of a high population density in Rwanda we

were able to have a random sample of over 400 households for farmers and consumers[4]. Subsequently, we interviewed over 2,100 households (farmers and consumers) across all three countries and analysed data using Stata/SE 16.0. However, other value chain analyses including key activities and how they are executed, challenges and opportunities at each actor segment, and market margins are presented in a technical report that we presented to the project steering committee and available upon request.

Table 1: Sample sizes across countries

Actor type	Rwanda (1,160)	DRC (809)	Ethiopia (593)
Inputs stockists	25	7	0
Farmers	520	281	190
Middlemen / traders	61	94	10
Processors	23	50	1
Wholesalers	12	22	0
Retailers	54	38	0
Consumers	465	314	392

We collected data using questionnaires with both closed and open-ended questions. Questions ranged from household biodata, demographics, agricultural production, food consumption, waste generation, income, to social attitudes and perceptions on CBE practices. Some questions were specific to certain actor segments. However, most questions, such as those on household demographics, consumption, awareness, knowledge, support for CBE practices, and opinion on eating CBE foods, were asked to all respondents. Household heads were the primary targets, and where unavailable, secondary decision makers were interviewed. Trained enumerators collected data electronically using open data kit (ODK) between August and December 2019.

Having an interest in understanding the CBE status quo with regards to policies, we also collected some policy data by reviewing potentially CBE related policies from government websites using keywords such as agriculture, environment, organic farming, composting, waste management etc. We cross-checked specific country policies using expert and key informant interviews administered by locally resident RUNRES scientists to our local partners, municipal authorities, and ministry staff including that of environment, agriculture, standards, industry, and trade. From local reviews added to online reviews, we realized that CBE policies are largely

[4] We used Rasoft software on http://www.raosoft.com/samplesize.html to confirm WFP sample recommendations if we aimed at a 95% confidence interval and a 5% error margin vis-à-vis city-region farmer populations

unavailable (DRC) and where available, were largely incomprehensive (Rwanda and Ethiopia). Hence, we did not investigate policies further but briefly comment on them here. For instance, in Rwanda, there is policy advocacy for organic composting, but use of human waste is prohibited due to potential health risks (RR, 2015; RR, 2017). In Ethiopia, policies promote the use of organic matter to improve soil structure and fertility. Utilization of on-farm forage, and fodder as animal feeds is also promoted. However, there are no regulations on use of human waste as fertilizer (FDRE, 1997; 2010). We present a summary of those CBE related policies we became aware of from both online, experts and key informant interviews in appendix Table A1.

2.3. Data Analysis

2.3.1. Descriptive analysis

We have used means or percentages of various variables generated in Stata to descriptively describe the status quo around CBE practices and CBE foods in target countries. The descriptive analysis gives us a summarized general data outlook on key outcome and explanatory variables.

2.3.2. Regression analysis

Because for instance, we are interested in understanding households' varying preferences (that are intrinsic but presented in a numerically meaningful way) for various CBE foods, based on assumptions that consumers make about these CBE foods, like safety, cultural acceptability, and others, we follow Fishburn (1968) and rely on the utility theory to model these preferences given a set of choices. More specifically, since our set of choices were merely rankings, that could only tell us what a consumer preferred more or less but not necessarily telling us the value attached to such preference, we assume the ordinal utility theory, (Debreu 1960; Tangian, 2004). The ordinal utility theory assumes that it is worth to only ask which choice is better than the other, but it is worthless to ask how much better a choice is or how good it is compared to others, (Tangian, 2004). Therefore, we follow an ordinal utility function to model farmers and consumers' preferences that are presented on an ordinal scale (strongly agree, agree, neutral, disagree, and strongly disagree). We do not derive the ordinal utility function to minimize space, but simply apply the concept. Hence, we subsequently estimated equation (1), as an ordered logistic regression since outcomes were ordinal, to enable us understand the likelihood of each ordinal outcome category, and the factors that could explain such outcomes (Greene 2009; Greene, 2012).

$$AKSF_i = \alpha + \beta \boldsymbol{X_i} + \partial \boldsymbol{Z_i} + \varepsilon_i \quad (1)$$

$AKSF_i$ was the ordinal outcome variable depicting at what level (strongly agree, agree, neutral, disagree, and strongly disagree) were households aware, knowledgeable, or supportive of CBE practices, or at what level would households accept to eat foods grown on compost, treated urine, or faecal matter. $\boldsymbol{X_i}$ is a vector of household characteristics, and $\boldsymbol{Z_i}$ is a vector of contextual factors including country effects; while $\alpha, \beta,$ and ∂ are parameters we estimated. ε_i is a random error. However, proportional odds logistic regression models as the ordered logit, must satisfy a key fundamental assumption requiring that no explanatory variable has a disproportionate effect on a particular outcome variable level / category. This is the proportional odds assumption. On testing data for this assumption as a validation step for our model, we found that our data violated this assumption, thus we re-estimated equation (1) as a generalized ordered logistic regression to correct this violation, (Long & Freese, 2014). Test results are presented below each results Table.

In our analysis, we selected as explanatory variables age, education, agricultural income, distance to nearest major town, severe food insecurity, marital status, gender, household size, and mobile phone use. Our choice is not only informed by field observations but also by literature, which we do not cite here to avoid redundancy, since we dominantly use the same in the discussions. Such literature and field observations guided us to the following general hypotheses: 1) households that are food insecure, married, or educated are likely to support CB practices, and willing to eat CB foods, 2) urban households are not likely to support CB practices, nor willing to eat CB foods, because there is currently heavy accumulation of waste in towns (GGGI, 2018; Krütli et al. 2018). In addition, because waste management is labour intensive (GGGI, 2018; Krütli et al. 2018), we hypothesized that married or larger sized households would support CBE practices, since they would have higher person numbers for labour. We present these variables in Table 2.

From Table 2, across all three city-regions, we observed that households are mostly male headed, married, with heads mostly attaining a primary school level for formal education. Household heads were, on average aged 45 years across the three city-regions. Household sizes ranged from 5 persons in Kamonyi-Rwanda to 8 in Bukavu-DRC. Most households used mobile phones. Agricultural income in Arba Minch-Ethiopia was nearly three times that of samples in Kamonyi or Bukavu. About half of the households in Bukavu experienced a form of severe food insecurity, while only 18% in Kamonyi and 5%in Arba Minch shared a similar experience. High

severe food insecurity in Bukavu may be due to persistent armed conflicts in eastern DRC, where our sample area belongs, (GCT, 2022).

For the regression results' interpretations, we prioritized associations of covariates for the strongly agree category, because interpreting all categories' results would be so bulky. Moreover, the strongly agree category is a very high standard that could more likely yield predictions of a higher possibility to be valid internally and externally. Nevertheless, we present all other results in respective Tables for readers' comparison. For continuous variables that showed a non-normal distribution, we followed Box and Cox (1964) to transform the affected variables, to yield residuals that were less heteroskedastic and approximately distributed normally. Boxcox transformations also corrected for average outliers, but for extreme outliers - for instance a respondent who appeared to have over 120 years, these were followed up physically through telephoning respondents for the correct data. Outliers could easily be detected with the tab command in Stata.

Table 2: Description of variables used in regressions

	Means					
Countries	DRC (N = 595)		Rwanda (N = 997)		Ethiopia (N = 583)	
Distance to nearest major town (km)	15.2	(15.7)	5.75	(5.61)	11.25	(7.77)
Education of head (years)	8.04	(5.99)	6.29	(3.91)	5.81	(5.52)
Household size (Persons)	7.69	(3.31)	5.12	(1.98)	5.75	(2.37)
Agriculture income/season (USD)	134.9	(217.2)	151.4	(275.4)	466.8	(936.1)
Age of head (years)	44.4	(15.7)	45.4	(12.1)	44.91	(16.6)
Severe food insecurity (score from 3 questions)	0.89	(1.07)	0.35	(0.84)	0.09	(0.42)
Percentages						
Male household head (D)	74		77		79	
Mobile phone use (D)	57		92		78	
Severe food insecurity experienced (D)	51		18		5	
Household head is married (D)	82		85		83	

In parentheses are standard deviations; USD is United States Dollar, 1USD = 1,607 Congolese francs = 930 Rwandese francs = 30 Ethiopian birr; D is dummy; Severe food insecurity refers to questions 7, 8, and 9 of (Household Food Insecurity Access (HFIAS) protocol

Regarding the outcome variables, we use the terms based on a generalized perception of respondents – where awareness was merely having heard of such a CBE practice, and knowledge was having any skill about how that CBE practice could be realized, and support was generally reflecting an opinion for if respondent would accept / recommend such a CBE practice to other community members. Because we focused on households that were cautiously using biological products and processes like fermentation to recycle and reuse organic waste (food, household, and human) with an intent of reducing waste and closing nutrient loops within their own food systems and improve their food systems' sustainability and productivity, we classified these practices as

CBE ones, and their food products as CBE foods, following (Geissdoerfer et al. 2017; Carus and Dammer, 2018; Kershaw et al. 2021; Muscat et al. 2021).

3. Results

3.1. *Descriptive results*

3.1.1. What CBE practices do currently exist in the respective farming systems?

In all three countries some CBE practices are prevalent (Table 3). For instance, most Rwandan farmers (77%) produce organic compost from crop residues and animal excreta and used this in crop production. In DRC, almost all farmers (99%) produce and use organic compost, but only about 3% of consumers[5] recycle waste and reuse it as compost or animal feed in urban agriculture. Also, most (75%) farmers in Ethiopia recycle crop residues or animal excreta and reuse it as organic compost.

Table 3: Current circular bioeconomy (CBE) practices

Country	Actor type	CBE practice (production and use of)	Percent age	Source of materials	Destination of CBE product
Rwanda	Farmers	Organic compost, animal feeds	77	Crop residues, cassava peels, and animal excreta	Crop production, livestock feeds
DRC	Farmers	Organic compost	99	Crop residues like coffee husks and animal excreta	Crop production
	Consumers	Organic compost, and animal feeds	3	Household organic, food, and human (urine) waste	Crop production and poultry feeds
Ethiopia	Farmers	Organic compost	75	Crop residues and animal excreta	Crop production

3.1.2. Are communities currently aware, knowledgeable, supportive of CBE concepts?

Most respondents stated that they were aware of, knowledgeable of, and/or supportive of CBE practices (Table 4). The least (55%) sample proportion for agreement (agree and strongly agree categories) to any CBE aspect (awareness, knowledge, or support) was in DRC, whereas this proportion was as high as 89% in Rwanda.

Table 4: Household awareness, knowledge, or support for CBE practices

	Percentages								
Countries	Rwanda (N=997)			DRC (N=595)			Ethiopia (N=583)		
Variables	Aware	Knowledge	Support	Aware	Knowledge	Support	Aware	Knowledge	Support
Strongly agree	16	14	21	17	17	16	39	32	31

[5] Consumer samples in all other countries never practiced CBE activities

Agree	72	74	68	47	44	39	41	48	43
Neutral	3	3	3	1	1	1	18	18	24
Disagree	7	7	6	35	38	4	1	1	1
Strongly disagree	2	2	2	0	0	0	1	1	1

3.1.3. What are communities' opinions about consuming foods grown on CBE fertilizers?

The stated acceptance to eat CBE foods is quite high across countries and more so for foods grown on organic compost (Figure 1). In Rwanda, about 91% would accept (agree + strongly agree) to eat CBE foods from organic compost, treated urine, or faecal material. These proportions are also relatively high in DRC (84%), and Ethiopia (72%). However, on eating CBE foods from human waste, there was disagreement (disagree + strongly disagree) of at least 8% across all countries. Moreover, sample proportions that were neutral (indifferent to accept or not accept) were also high, reaching about 22% in Ethiopia for CBE foods from urine and faecal fertilizers.

Figure 1: Household opinion on eating foods grown on CBE fertilizers

3.2. Regression results

3.2.1. Which factors do explain communities' CBE awareness, knowledge, and support?

Results in Table 5 indicate that households that lived farther from major towns, were less likely by a likelihood of 0.6% to strongly agree to be aware of CBE practices. On the other hand, households whose heads used mobile phones or were married, or had larger family sizes, or had higher incomes, or were older, were more likely by a likelihood of 4.8%, 5.2%, 0.8%, 0.4%, and 0.2% respectively to strongly agree to be aware of CBE practices. Considering country specific effects, households in Rwanda and Ethiopia compared to those in DRC, were more likely by a likelihood of 13% and 46% respectively to strongly agree to be aware of CBE practices.

Table 5: Marginal effects after generalized ordered logit for awareness on CBE practices

**Model Outcomes:** Stated Awareness levels	Explanatory Variables									
	Distance to major town (km)	Male household head (D)	Education of head (years)	Mobile phone use (D)	Household size (Persons)	Agric. income /season (USD)	Age of head (years)	Severe food insecurity (score)	Household head is married (D)	Country	
										Rwanda	Ethiopia
(1) **Strongly agree** *Pr=0.175****	-0.006*** (0.001)	-0.024 (0.023)	0.002 (0.002)	0.048** (0.021)	0.008** (0.004)	0.004*** (0.001)	0.002*** (0.001)	-0.013 (0.010)	0.052** (0.024)	0.128*** (0.012)	0.461*** (0.032)
(2) Agr**ee** *Pr=0.723****	0.005*** (0.002)	-0.004 (0.027)	0.006*** (0.002)	-0.06** (0.023)	-0.003 (0.004)	-0.001 (0.001)	-0.002** (0.001)	0.005 (0.011)	0.003 (0.031)	-0.26*** (0.019)	-0.62*** (0.034)
(3) **Neutral** *Pr=-0.22****	0.003** (0.001)	0.024** (0.011)	-0.0002 (0.001)	0.015 (0.026)	-0.001 (0.002)	-0.001** (0.001)	$-4x10^{-5}$ (0.0004)	0.017*** (0.006)	-0.045** (0.019)	1.001*** (0.029)	1.122*** (0.027)

(4) **Disagree**	-0.001	-0.006	-0.006	0.008	0.0001	0.0001	-7×10^{-6}	0.125	-0.006	-1.026	-1.414
Pr=0.301	(0.009)	(0.723)	(0.0467)	(41.20)	(0.341)	(0.141)	(0.071)	(12.70)	(0.340)	(10.64)	(22.06)
(5) **Strongly**	-0.0001	0.008	-0.001	-0.012	-0.004	-0.002	-0.001	-0.135	-0.004	0.151	0.453
**disagree**	(0.009)	(0.723)	(0.047)	(41.20)	(0.341)	(0.141)	(0.073)	(12.70)	(0.340)	(1.129)	(12.95)
Pr=0.019											

*Standard errors in parentheses; *** p<0.01, ** p<0.05, * p<0.1; Pr. is probability for the given outcome when all predictors are at mean values; D is dummy, USD is United States Dollars, KM is kilometers; Approximated likelihood ratio test of proportionality of odds across response categories after ordered logit, Chi2 =621.52***; Generalized Ordered Logit estimates: Pseudo R2 = 0.396, Chi2 = 1,865.54***, Observations = 2,175*

Results in Table 6, indicate that households that lived farther from towns, or those that suffered severe food insecurity were less likely by a likelihood of 0.2%, and 0.5% respectively, to strongly agree to be knowledgeable about CBE practices. On the other hand, households with more educated heads, or those with larger family sizes, or had higher agricultural income, or older heads, or married heads were more likely by a likelihood of 0.6%, 0.7%, 0.2%, 0.3%, and 5% respectively to strongly agree to be knowledgeable about CBE practices. However, households in Rwanda and Ethiopia compared to those in DRC were more likely by a likelihood of 11%, and 23% respectively to strongly agree to be knowledgeable about CBE practices.

Table 6: Marginal effects after generalized ordered logit for knowledge on CBE practices

**Model outcomes:** Stated Knowledge levels	Explanatory Variables									
	Distance to major town (km	Male household head (D)	Education of head (years)	Mobile phone use (D)	Household size (Person)	Agric. income/ season (USD)	Age of head (years)	Severe food insecurity (score	Household head is married (D)	Country	
										Rwanda	Ethiopia
(1) **Strongly agree**	-0.002^{*}	-0.019	0.006^{***}	0.021	0.007^{***}	0.002^{***}	0.003^{***}	-0.005^{**}	0.053^{***}	0.109^{***}	0.233^{***}
	(0.001)	(0.015)	(0.001)	(0.014)	(0.002)	(0.0003)	(0.0004)	(0.002)	(0.014)	(0.014)	(0.019)
Pr=0.152***											
(2) **Agree**	-0.0003	-0.003	0.001^{***}	0.006	0.001^{**}	0.0004^{***}	0.001^{***}	-0.001^{*}	0.024^{**}	0.157^{***}	0.118^{***}
Pr=0.625***	(0.0002)	(0.002)	(0.0004)	(0.005)	(0.001)	(0.0001)	(0.0002)	(0.001)	(0.010)	(0.024)	(0.026)
(3) **Neutral**	0.001^{*}	0.005	-0.002^{***}	-0.006	-0.002^{**}	-0.001^{***}	-0.001^{***}	0.001^{**}	-0.017^{***}	-0.055^{***}	-0.083^{***}
Pr=0.069***	(0.0003)	(0.004)	(0.0004)	(0.004)	(0.001)	(9×10^{-5})	(0.0001)	(0.001)	(0.005)	(0.008)	(0.008)
(4) **Disagree**	0.001^{*}	0.015	-0.005^{***}	-0.019	-0.01^{***}	-0.002^{***}	-0.002^{***}	0.004^{**}	-0.054^{***}	-0.193^{***}	-0.247^{***}
Pr=0.143***	(0.001)	(0.012)	(0.001)	(0.013)	(0.002)	(0.0003)	(0.0004)	(0.002)	(0.017)	(0.027)	(0.023)
(5) **Strongly disagree**	0.0001	0.002	-0.001^{***}	-0.002	-0.001^{**}	-0.0002***	-0.0002^{**}	0.0004^{*}	-0.006^{***}	-0.018^{***}	-0.021^{***}
	(9×10^{-5})	(0.001)	(0.0001)	(0.001)	(0.0002	(4×10^{-5})	(6×10^{-5})	(0.0002)	(0.002)	(0.005)	(0.005)
Pr=0.011***											

*Standard errors in parentheses; *** p<0.01, ** p<0.05, * p<0.1; Pr. is probability for the given outcome when all predictors are at mean values; D is dummy, USD is United States Dollars, KM is kilometers; Approximated likelihood ratio test of proportionality of odds across response categories, after ordered logit, Chi2 =576.01***; Generalized Ordered Logit estimates: Pseudo R2 = 0.379, Chi2 = 1,787.41***, Observations = 2,175*

From Table 7, results indicate that households that lived farther from towns, or those that suffered severe food insecurity, were less likely by a likelihood of 1%, and 0.4% respectively to strongly agree to support CBE practices. However, households whose heads used mobile phones, or had higher agricultural incomes, or were older, were more likely by a likelihood of 4.7%, 0.4%, and 0.3% respectively to strongly agree to support CBE practices. Analysis of country effects, indicated that households in Rwanda and Ethiopia, compared to those in DRC, were more likely by a likelihood of 19%, and 42% respectively to strongly agree to support CBE practices.

Table 7: Marginal effects after generalized ordered logit for support on CBE practices

**Model Outcomes:** Stated Support levels	Distance to major town (km	Male household head (D)	Education of head (years)	Mobile phone use (D)	Household size (Person)	Agric. income/ season (USD)	Age of head (years)	Severe food insecurity (score)	Household head is married (D)	Country: Rwanda	Country: Ethiopia
(1) **Strongly agree**	-0.01^{***}	-0.024	-0.003	0.047^{**}	0.006	0.004^{***}	0.003^{***}	-0.037^{***}	0.040	0.194^{***}	0.415^{***}
$Pr=0.178^{***}$	(0.002)	(0.024)	(0.002)	(0.022)	(0.004)	(0.001)	(0.001)	(0.012)	(0.025)	(0.019)	(0.034)
(2) **Agree**	0.005^{***}	-0.020	0.009^{***}	-0.06^{**}	$-9x10^{-5}$	-0.001	-0.001^{**}	0.027^{**}	0.011	-0.33^{***}	-0.640^{***}
$Pr=0.695^{***}$	(0.002)	(0.025)	(0.002)	(0.024)	(0.004)	(0.001)	(0.001)	(0.013)	(0.031)	(0.023)	(0.036)
(3) **Neutral**	0.002	0.043^{***}	0.001	0.013	-0.008^{***}	-0.002^{***}	$-6x10^{-5}$	0.019^{***}	-0.039^{*}	1.024^{***}	1.193^{***}
$Pr=-0.19^{***}$	(0.001)	(0.012)	(0.002)	(0.023)	(0.003)	(0.001)	(0.001)	(0.007)	(0.022)	(0.021)	(0.024)
(4) **Disagree**	-0.002	-0.005	-0.006	0.003	0.004	0.0003	-0.0002	0.102	-0.003	-1.039	-1.427
Pr=0.303	(0.002)	(0.569)	(0.085)	(32.18)	(0.163)	(0.096)	(0.074)	(11.46)	(0.787)	(13.18)	(28.19)
(5) **Strongly disagree**	$-2x10^{-5}$	0.006	-0.001	-0.002	-0.002	-0.001	-0.001	-0.111	-0.009	0.147	0.460
Pr=0.015	(0.002)	(0.569)	(0.085)	(32.18)	(0.163)	(0.096)	(0.074)	(11.46)	(0.787)	(0.887)	(16.62)

*Standard errors in parentheses; *** p<0.01, ** p<0.05, * p<0.1; Pr. is probability for the given outcome when all predictors are at mean values; D is dummy, USD is United States Dollars, KM is kilometers; Approximated likelihood ratio test of proportionality of odds across response categories, after ordered logit, Chi2 =680.71***; Generalized Ordered Logit estimates: Pseudo R2 = 0.338, Chi2 = 1,983***, Observations = 2,175*

In brief, households that stayed farther from towns, or those that suffered severe food insecurity were significantly less likely to strongly agree to be aware, knowledgeable, or supportive of CBE practices. The opposite was true for households whose heads were married, better educated, older, used mobile phones, or had better agricultural income. At country level, Rwandan and Ethiopian households compared to those in DRC, were more likely to be aware, knowledgeable, and supportive of CBE practices – with greater effects being observed in Ethiopia.

3.2.2. What factors explain households' opinion of "strongly agree" to eat CBE foods?

From Table 8, households that lived farther from towns were less likely by a likelihood of 0.7% to strongly agree to eat food grown on compost. However, households whose family sizes were bigger, or those whose heads used mobile phones, or were older, were more likely by a likelihood of 0.9%, 6%, and 0.2% respectively to strongly agree to eat foods grown on compost. Country effects indicate that Rwandan and Ethiopian households compared to DRC ones, were less likely by 18%, and 28% respectively to strongly agree to eat foods grown on compost.

Table 8: Marginal effects after generalized ordered logit on eating foods grown on compost

**Model Outcomes:** Could accept eat Compost grown food	Distance to major town (km	Male household head (D)	Education of head (years)	Mobile phone use (D)	Household size (Person)	Agric. income/ season (USD)	Age of head (years)	Severe food insecurity (score)	Household head is married (D)	Country: Rwanda	Country: Ethiopia
(1) **Strongly agree**	-0.007^{***}	-0.004	0.001	0.061^{**}	0.009^{**}	0.001	0.002^{***}	-0.014	0.024	-0.18^{***}	-0.280^{***}
$Pr=0.512^{***}$	(0.001)	(0.029)	(0.002)	(0.029)	(0.004)	(0.001)	(0.001)	(0.014)	(0.035)	(0.034)	(0.032)
(2) **Agree**	0.005^{***}	0.010	0.001	-0.06^{**}	-0.005	0.001^{*}	-0.002^{***}	0.017	-0.011	0.168^{***}	0.079^{**}
$Pr=0.421^{***}$	(0.001)	(0.030)	(0.002)	(0.029)	(0.004)	(0.001)	(0.001)	(0.015)	(0.035)	(0.034)	(0.034)

(3) **Neutral**	0.003^{***}	0.001	-0.003^{***}	-0.007	-0.004^{**}	-0.002^{***}	$-3x10^{-5}$	-0.004	-0.018	0.009	0.103^{***}
Pr=0.041***	(0.001)	(0.013)	(0.001)	(0.011)	(0.002)	(0.001)	(0.0003)	(0.008)	(0.014)	(0.007)	(0.020)
(4) **Disagree**	-0.001	-0.007	-0.001	-0.011	-0.007	0.001	-0.0004	-0.011	0.024	-0.003	-0.019
Pr=0.010	(0.001)	(0.011)	(0.001)	(0.012)	(0.005)	(0.001)	(0.0003)	(0.008)	(0.039)	(0.004)	(0.078)
(5) **Strongly**	-0.0002	-0.001	0.001	0.018^{**}	0.007	-0.001	0.0001	0.012^{**}	-0.019	$2x10^{-7}$	0.118
**disagree**	(0.001)	(0.009)	(0.001)	(0.008)	(0.005)	(0.001)	(0.0003)	(0.005)	(0.040)	(0.0002)	(0.076)
Pr=0.016**											

*Standard errors in parentheses; *** p<0.01, ** p<0.05, * p<0.1; Pr. is probability for the given outcome when all predictors are at mean values; D is dummy, USD is United States Dollars, KM is kilometers; Approximated likelihood ratio test of proportionality of odds across response categories, after ordered logit, Chis2 =183.89***; Generalized Ordered Logit estimates: Pseudo R2 = 0.099, Chi2 = 410.15***, Observations = 2,160*

From Table 9, households that lived farther from towns, or those which suffered severe food insecurity, or those whose heads were more educated, were less likely by a likelihood of 0.9%, 3.1%, and 0.5% respectively to strongly agree to eat foods grown on treated urine. On the other hand, households with larger family sizes, or those whose heads used mobile phones, or had higher agricultural income, or their heads were older, or married, were more likely by a likelihood of 0.7%, 6%, 0.3%, 0.3%, and 5.5% respectively to strongly agree to eat foods grown on treated urine. However, households in Rwanda and Ethiopia compared to those in DRC, were less likely by a likelihood of 19%, and 15% respectively to strongly agree to eat foods grown on treated urine.

Table 9: Marginal effects after generalized ordered logit on eating foods grown on treated urine

**Model**	Explanatory Variables										
**Outcomes:** Could eat treated Urine grown food	Distance to major town (km)	Male household head (D)	Education of head (years)	Mobile phone use (D)	Household size (Person)	Agric. income/ season (USD)	Age of head (years)	Severe food insecurity (score)	Household head is married (D)	Country	
										Rwanda	Ethiopia
(1) **Strongly**	-0.009^{***}	0.019	-0.005^{**}	0.060^{**}	0.007^{*}	0.003^{***}	0.003^{***}	-0.031^{**}	0.055^{*}	-0.19^{***}	-0.150^{***}
**agree**	(0.001)	(0.026)	(0.002)	(0.025)	(0.004)	(0.001)	(0.001)	(0.013)	(0.029)	(0.032)	(0.032)
Pr=0.285***											
(2) **Agree**	0.005^{***}	-0.018	0.010^{***}	-0.035	-0.002	0.001	-0.002^{**}	0.009	0.008	0.187^{***}	0.047
Pr=0.507***	(0.001)	(0.029)	(0.002)	(0.028)	(0.004)	(0.001)	(0.002)	(0.014)	(0.034)	(0.034)	(0.034)
(3) **Neutral**	0.002^{**}	0.022	-0.004^{***}	-0.016	-0.008^{***}	-0.002^{***}	-0.0002	0.019^{**}	-0.018	0.008	0.122^{***}
Pr=0.109***	(0.001)	(0.016)	(0.001)	(0.018)	(0.003)	(0.0004)	(0.001)	(0.008)	(0.021)	(0.019)	(0.022)
(4) **Disagree**	0.002^{***}	-0.014	-0.002	-0.016	0.002	-0.001^{***}	-0.001^{**}	-0.004	-0.025	-0.013	-0.032^{*}
Pr=0.077***	(0.001)	(0.016)	(0.001)	(0.015)	(0.002)	(0.0004)	(0.0004)	(0.007)	(0.019)	(0.021)	(0.019)
(5) **Strongly**	0.001^{**}	-0.009	0.001	0.007	0.001	-0.0004	$2x10^{-5}$	0.005	-0.020	0.008	0.012
**disagree**	(0.001)	(0.010)	(0.001)	(0.008)	(0.001)	(0.0003)	(0.0003)	(0.004)	(0.014)	(0.013)	(0.012)
Pr=0.023***											

*Standard errors in parentheses; *** p<0.01, ** p<0.05, * p<0.1; Pr. is probability for the given outcome when all predictors are at mean values; D is dummy, USD is United States Dollars, KM is kilometers; Approximated likelihood ratio test of proportionality of odds across response categories, after ordered logit, Chi2 =95.94***; Generalized Ordered Logit estimates: Pseudo R2 = 0.059, Chi2 = 315.05***, Observations = 2,160*

From Table 10, households that lived farther from towns, or those that suffered severe food insecurity, or those whose heads were more educated, were less likely by a likelihood of 0.9%, 3.4%, and 0.7% respectively to strongly agree to eat foods grown on faecal matter. On the other hand, households whose heads used mobile phones, or were older, or had higher agricultural incomes, or were married, were more likely by a likelihood of 4.8%, 0.3%, 0.3%, and 6.3% respectively, to strongly agree to eat foods grown on faecal matter. Again, households in Rwanda,

and Ethiopia were less likely by a likelihood of 17% and 23% respectively to strongly agree to eat foods grown on faecal matter, compared to those in DRC.

Table 10: Marginal effects after generalized ordered logit on eating foods grown on faecal matter

**Model Outcomes:** Could eat fecal matter grown food	Explanatory Variables										
	Distance to major town (km	Male household head (D)	Education of head (years)	Mobile phone use (D)	Household size (Person)	Agric. income/ season (USD)	Age of head (years)	Severe food insecurity (score)	Household head is married (D)	Country	
										Rwanda	Ethiopia
(1) **Strongly agree**	-0.009***	0.021	-0.007***	0.048*	0.003	0.003***	0.003***	-0.034***	0.063**	-0.17***	-0.226***
Pr=0.287***	(0.002)	(0.026)	(0.002)	(0.025)	(0.004)	(0.001)	(0.001)	(0.013)	(0.029)	(0.033)	(0.031)
(2) **Agree**	0.004***	0.019	0.011***	-0.037	$-7x10^{-5}$	0.001	-0.001	0.010	-0.028	0.200***	0.085**
Pr=0.513***	(0.001)	(0.029)	(0.002)	(0.027)	(0.004)	(0.001)	(0.001)	(0.014)	(0.033)	(0.034)	(0.034)
(3) **Neutral**	0.001	0.005	-0.002*	-0.019	-0.005*	-0.001***	-0.0002	0.013*	-0.0004	-0.047**	0.157***
Pr=0.098***	(0.001)	(0.013)	(0.001)	(0.016)	(0.003)	(0.0004)	(0.001)	(0.007)	(0.016)	(0.019)	(0.023)
(4) **Disagree**	0.003***	-0.036**	-0.002*	0.002	$-5x10^{-5}$	-0.002***	-0.001***	0.004	-0.015	-0.002	-0.027
Pr=0.076***	(0.001)	(0.015)	(0.001)	(0.014)	(0.002)	(0.0004)	(0.0004)	(0.006)	(0.018)	(0.022)	(0.019)
(5) **Strongly disagree**	0.001**	-0.009	0.0003	0.006	0.002	-0.001*	$-4x10^{-5}$	0.007*	-0.020	0.014	0.011
Pr=0.026***	(0.001)	(0.009)	(0.001)	(0.008)	(0.002)	(0.0003)	(0.0003)	(0.004)	(0.013)	(0.015)	(0.014)

*Standard errors in parentheses; *** p<0.01, ** p<0.05, * p<0.1; Pr. is probability for the given outcome when all predictors are at mean values; D is dummy, USD is United States Dollars, KM is kilometers; Approximated likelihood ratio test of proportionality of odds across response categories, after ordered logit, Chi2 =204.30***; Generalized Ordered Logit estimates: Pseudo R2 = 0.080, Chi2 = 423.12***, Observations = 2,160*

In brief, households that lived farther from towns, and those that suffered severe food insecurity, or had more educated heads, were significantly less likely to strongly agree to eat CBE foods especially those grown on treated urine and faeces. On the other hand, households that had larger family sizes, with heads who used mobile phones, or were older, or married, or had higher agricultural incomes, were significantly more likely to eat CBE foods. However, households in Rwanda and Ethiopia compared to those in DRC, were significantly less likely to eat CBE foods. These country effects were larger in magnitude for Ethiopia than Rwanda.

4. Analysis and Discussion

4.1. Descriptive results

Across all food systems, farmers practice CBE concepts, yet, massive accumulation of waste is abundant in towns inhabited mostly by consumers (Kaza et al. 2018; Krütli et al. 2018; RUNRES, 2019; GGGI, 2018; 2019). After harvesting, farmers leave crop residues on farm to decompose into compost and replenish soil nutrients. However, there are no proper mechanisms used by farmers to guarantee quality of such compost. Therefore, the effectiveness of such practices is not clear. Moreover, farmers have certain crops that they prefer on which to apply this

compost. This implies that farmers' interests to practice CBE concepts could be driven by farm/crop typologies, but not necessarily that farmers view CBE practices as such a novel model to optimize resource use. Therefore, knowledge components would be important for CBE innovations to solidify community interests in overall CBE practices. Some farmers in Rwanda also use, in addition to field residues, other organic wastes as compost (for instance faecal matter sourced from pit latrines) or animal feeds (for instance cassava peels), implying that innovations that valorise organic waste into compost or animal feeds could stand a higher chance of success among farmers, since farmers could provide a ready market for these CBE products.

High levels of awareness, knowledge, and support for CBE practices across all city-regions suggest a high chance for success of CBE based innovations in these city-regions, and others with similar food systems. This is because these attributes are highly correlated within city-regions. For instance, in Rwanda the Spearman correlation for Awareness (A), Knowledge (K) and Support (S) was A-K = 0.88, A-S = 0.85, K-S = 0.87. For DRC: for, A-K = 0.76, A-S = 0.83, K-S = 0.88. In Ethiopia the results were: A-K = 0.68, A-S = 0.51 and K-S = 0.64. However, the extent (level) of this awareness, knowledge, or support is not yet studied, and thus not known. The high proportions of each attribute in Rwanda could be attributed to a government focused on social and economic developments for nearly three decades (RR, 2017). However, high proportions of disagreement in DRC for CBE support highlight the need to further investigate the basis of such attitudes because they hamper optimal scaling of CBE innovations. For instance, there could be an inherent, negative social-cultural perception towards CBE practices among DRC communities, or simply that the instability due to persistent armed civil conflicts in eastern DRC haven't allowed effective community sensitization on CBE practices. Furthermore, understanding reasons[6] behind such a negative opinion could guide appropriate behavioural change interventions. The high possibilities of acceptance to eat CBE foods (figure 2) could also imply that most consumers would be comfortable eating CBE foods. Moreover, Vogel (2020) found that 96% and 86% of farmers in Rwanda would accept organic and human waste recycling practices respectively.

4.2.Regression results

[6] Our study did not investigate reasons for given consumption opinions

The strong relationship between education and CBE knowledge (Table 6), suggests that with proper knowledge, households could understand the usefulness of CBE practices which is essential for scaling out CBE practices. With better education, persons gain better capacity to understand concepts around which livelihoods are sustained, (Evans et al. 2012; Kirchherr & Piscicelli, 2019). This understanding builds trust for such concepts and subsequently acceptance of their products (Hazen et al. 2016). Alternatively, with better education, households can easily understand the associated health risks around CBE foods from human excreta, (Lwin et al. 2017). Currently, urine and faeces are only stored for some time for instance 3 months before being applied on plants, where farmers assume that this is sufficient time to kill pathogens. Current products have not been tested at all to certify their safety. Our results are similar to those of Wu et al. (2012), and McCarthy et al. (2016) who found highly educated consumers more willing to eat certified, safer, and healthier foods. Moreover, some foods for instance cassava, it is the roots which directly ingest CBE fertilizers, that are eaten and in case that the treatment was not effective, this could be dangerous to consumers. Hence, to increase acceptance of CBE foods, CBE innovations must be able to prove the safety of CBE products so to motivate consumer confidence, through effective CBE knowledge and learning components. Interestingly, for instance the Rwanda Standards Board, has embarked on working with the private sector developing CBE innovations, on the necessary safety standards to be observed before formally availing on the market any CBE products especially those from human excreta.

Agriculture is the major livelihood source for most households in studied countries. But because farm inputs are expensive, dependence on agriculture motivates and equips farmers with indigenous knowledge to practice CBE, probably due to a lack of other alternatives. Moreover, within considered countries, recycling of organic waste for reuse in agriculture is largely promoted by governments (RR, 2015; 2017; DRC, 2011; FDRE, 1997; 2010). Furthermore, most African food systems rely on naturally available resources and cultivate with minimal farm inputs (Pelster et al. 2017). Hence, composting is one natural way used by farmers to replenish soil nutrients. Thus, CBE innovations could target agricultural households especially those that earn better incomes from agriculture, for demand of CBE products like compost. Households with better agricultural incomes were also more likely to accept eat CBE foods. Our findings are similar to those of Fleischhacker et al. (2011), and French et al. (2019), who found high-income households to eat healthier and specialized food. CBE foods require resources (time, labour, and money) that

may be incurred in recycling, packaging, transportation, and application of CBE fertilizers; thus, CBE foods could currently be comparatively expensive. Therefore, CBE innovations must design and develop cost-effective CBE practices that should yield cost-effective products, and thus CBE foods accessible to the poor. Nevertheless, we acknowledge that there may be other organic material other than that considered here, which could even be more cost-effective to recycle and reuse – for instance animal (dung) waste, but such other organic waste was not readily available to most households that we interviewed. Also, we acknowledge, that CBE processes currently could be cost-ineffective since compost making requires some costs, (Jara-samaniego et al. 2017). However, we believe that if CBE fertilizers are used on high commercial-value crops like vegetables, ornaments, or coffee where high-value products can be generated and sold at competitive global markets, the entire process can ultimately produce net positive revenues. Moreover, composting has been found to have potential for commercial viability, (Abdul Rahman et al. 2020; Malik and Rawat, 2021; Gatune et al. 2021). Since we focussed on exploring existence of CBE practices and community preferences about their products, we do not yet assess CBE commercial viability in the selected city-regions, in this paper, but in the technical report we found that processors sold organic waste for money.

The inverse relationship between increasing household distance to major towns and the likelihood to strongly be aware, knowledgeable, or supportive of CBE practices, or acceptance to eat CBE grown foods, implies that peri-urban and urban consumers would be the more likely market for CBE innovations and CBE foods. CBE practices are intensive in time, labour, and money which could be difficult to apply over relatively larger farm areas as those in rural areas, but handy for averagely small farm areas in peri-urban or urban agriculture. Our findings are similar to those of Mohamad et al. (2014), who found urban consumers in Malaysia to be more aware of organic foods, and readily ate such food. Also, the increasingly active sustainability and food safety standards that are more visible in urban settlements, give urban residents more confident in foods from urban markets. Moreover, rural areas are inhabited by communities strong in traditional beliefs, which may hinder acceptance for CBE foods especially that grown on human excreta (Kyomuhendo 2003; Appiah-Opoku 2007). Also, rural communities are less exposed to daily food insecurity, as these may have more land resources to grow foods, (Njuki et al. 2011).

Severe food insecurity was associated with less awareness, knowledge, and support for CBE practices (Table 5, 6, and 7). This is in agreement with Nata et al. (2014), who found food insecurity to be associated with low support for soil-improving practices, that are similar to CBE practices. Severe food insecurity could partly be leading to the numerous armed conflicts in eastern DRC, with about a hundred waring groups, thus bringing about an environment where knowledge on developmental innovations like CBE can hardly be exchanged, (GCT, 2022). Therefore, CBE innovations could need to prioritize household food security to succeed. For example, CBE innovations could improve animal/crop productivity to directly tackle food insecurity, or yield products that are economically viable to enhance income, and thus improve food security via income pathways. Literature notes that men are more likely to participate in agriculture where there are financial gains (Njuki et al. 2011). However, composting, the most promoted CBE practice observed, is yet to be reasonably commercialized (RR, 2015; 2017; DRC, 2011; FDRE, 1997; 2010; FAO, 2013) and could explain the negative likelihood for CBE support among male headed households (Table 7). Thus, CBE innovations should prioritize commercial viability of CBE products to attract male headed (majority) households. Surprisingly, households with severe food insecurity were less likely to eat CBE foods, which could still point to possible perceived health risks given the current methods of producing CBE fertilizers, or merely strong negative social-cultural values, yet these need longer time to change (Inglehart & Baker, 2000). In some instances, social stigma has been found to negatively influence food acceptance by food insecure persons (Pogash, 2008; Purdam et al. 2015). Therefore, CBE innovations must be aware of potential social dislikes, and strengthen awareness campaigns on safety, and appropriateness of CBE foods. Wrong consumer perceptions may generate social stigma against CBE foods.

Interestingly, we found that married households also had a higher likelihood to be aware and knowledgeable of CBE practices (Table 5, and 6), and as well accept to eat CBE foods (Table 9, 10). Married households usually have children, hence, are likely to face higher household food insecurity challenges due to having more persons to feed, as did find Kaza et al. (2018) and Sulaiman et al. (2021). Hence, resorting to resource conservative foods[7], such as CBE foods, is logical. For instance, composting has been found to be commercially viable in Asia by, (Abdul Rahman et al. (2020) and Malik and Rawat, (2021), implying that it is resource use cost-effective.

[7] Readily available organic waste which makes raw materials cheaper, could enable better access to inputs like compost

Nevertheless, composting also bares some costs, (Jara-samaniego et al. 2017). Unfortunately, we don't ascertain costs related to each CBE practice in this study – given our study's limited scope and data. Our interpretation above is also supported by our results (Table 6, 8, and 9) where households with larger family sizes were more likely to be knowledgeable of CBE practices, and as well accept to eat CBE foods, especially those grown on compost and treated urine, that have more social acceptability than those grown on faeces. Therefore, CBE innovations could prioritize households with married heads, and those with larger family sizes. Furthermore, households with older heads were also more likely to be aware, knowledgeable, and supportive of CBE practices, and as well accept to eat any kind of CBE foods. As household heads age, they do apparently accumulate knowledge of the benefits of CBE practices, and subsequently accept CBE foods. This is further supported by Babaei et al. (2015), who found older household heads (over 45 years) more likely to recycle waste. Thus, CBE innovations could target households with heads of average old working age, as these could even be more likely to accommodate behavioural changes.

More interestingly, our analysis unearths important associations about household heads having mobile phones, and CBE practices and foods. Mobile phones foster awareness, exchange of information, access to knowledge and financial services (remittances and normal payments for especially financially excluded farm households), and maintenance of connectivity among social networks. This is consistent with Sekabira and Qaim, (2017b), Munyegera and Matsumoto, (2018), and Mukong and Nanziri, (2021) . Moreover, Kiconco et al. (2021) adds that social networks are a formidable source of knowledge, physical and financial support that could be essential in driving acceptance for CBE innovations, and CBE products. Therefore, CBE innovations could prioritize households with mobile phones to enhance information exchange and knowledge delivery.

Finally, the intriguing country effects show that households in Rwanda and Ethiopia were consistently more likely compared to those in DRC to be aware, knowledgeable, and supportive of CBE practices, however the reverse was true regarding accepting to eat CBE foods. This may be explained by the broader political economy aspects. Prior to early 2021 when data for this study was collected, Rwanda and Ethiopia had enjoyed decades of political stability with no civil wars unlike DRC. In fact, eastern DRC, where data was collected, has been suffering from frequent civil wars with over 20,000 United Nations peacekeepers, (GCT, 2022). Such unrests, have disrupted agriculture and other development and bred poverty and hunger, while developmental socio-

economic programs were uninterruptedly being rolled-out in Rwanda and Ethiopia, (RR, 2015; 2017). However, the higher likelihood of not accepting CBE foods in Rwanda and Ethiopia, could be linked to perceived health risks, or negative culture values.

4.3. Study limitations

The relatively high consistency of our results in direction and magnitude of effects across city-regions gives us confidence that our results are reliable. Moreover, we used native enumerators who could make logical interpretations of questions in local contexts around such a relatively new CBE concept in surveys. However, using only cross-section data with stated other than observed attributes and opinions is a limitation. We also did not ask respondents reasons for their attributes or opinions. We also exclusively used categorical data for dependent variables, which may not provide tangible unit effects. Nevertheless, given that CBE literature in Sub Sahara Africa (SSA) is largely scanty, we had to start somewhere to fill the knowledge gap. We recommend that experimental or panel data could be used in future to support this initial research. That said, we are confident that our results reflect the CBE status quo in studied city-regions and can reliably inform successful design, implementation and scaling of CBE innovations. However, given that this was an exploratory study – with several key aspects around CBE innovations not investigated, based on our findings, we suggest hypotheses to be investigated by future research around CBE innovations as below:

I. Prioritizing knowledge and learning components is essential for uptake of CBE innovations.
II. Targeting agricultural households guarantees demand for CBE products like compost animal feeds
III. Prioritizing household food security ensures uptake of CBE innovations
IV. Commercial viability of CBE innovations influences household adoption of CBE practices.
V. Targeting households with heads who use mobile phones, or those of average old working age, or those who are married, or those with better education, enables uptake of CBE innovations.
VI. Ensuring certification, safety, and sustainability standards of CBE products, increases demand for CBE products.

VII. Strengthening awareness on safety standards, and appropriateness of CBE foods for consumption, alleviates potential social and cultural dislikes against CBE foods.

VIII. Wrongful consumer perceptions about CBE foods generates social stigma against CBE foods

IX. Enacting comprehensive CBE policies enables faster deployment and scaling of CBE innovations

5. Conclusion

Using data from about 2,100 households, we analysed the status quo of CBE in some African food systems. We studied key food commodity value chains (banana in Ethiopia, coffee in DRC, and cassava in Rwanda), to analyse the awareness, knowledge, or support for CBE practices among farmers and consumers in these chains. We assessed opinions of respondents on consumption of CBE foods, and reviewed country policies on CBE practices. Farmers in all countries, and negligible proportions of DRC urban consumers practiced composting. Therefore, nutrient loops are still wide open in the rural-urban nexus. Furthermore, policies concerning circular bioeconomy appear to be insufficient, and thus need immediate attention for formulation, clarity, and enforcement. Nevertheless, our study households were largely aware, knowledgeable, and supportive of CBE practices. Moreover, the majority would accept to eat CBE foods and thus potential demand for CBE foods could exist especially in towns – more so higher income consumers, that would also be interested in organic foods. However, CBE innovations would benefit from the incorporation of a strong learning component to deliver knowledge, primarily targeting households with older or married heads or those with better education, or those that own mobile phones for ease of knowledge transfer. A focus on rural households heavily reliant on agriculture (with better agricultural incomes) as a market for compost, and urban consumers as market for CBE foods could render CBE innovations economically viable. However, to facilitate cheaper return of compost to rural areas, bulky fresh organic waste processing must happen near dumpsites in towns, and lighter dry processed compost returned to rural areas. CBE innovations could also need to prioritize household food security to attract sustainable community, and government support for CBE practices. CBE policies must be urgently and comprehensively formulated to guide proper design, development, and implementation of CBE innovations.

Acknowledgements: The authors extend their gratitude to the Swiss Agency for Development and Cooperation (SDC) for funding RUNRES under grant no: 7F09521. The authors are also thankful to all RUNRES scientists, enumerators, and staff of RUNRES partners that have contributed to data collection, and technical guidance in writing this article.

References

Abdul Rahman, H. M., Sadi, T., Ahmad, A. A., et al. (2020). Inventory and composting of yard waste in Serdang, Selangor, Malaysia, *Heliyon*, 6, (7): e04486, https://doi.org/10.1016/j.heliyon.2020.e04486.

Ambisa Z., Tesfa B., Olani T., and Abdeta D. (2019). Review on the Production and Marketing of Banana in Ethiopia. World *J. of Agriculture & Soil Science*, 2(1): 2019. doi: 10.33552/WJASS.2019.02.000529

Appiah-Opoku S. (2007). Indigenous Beliefs and Environmental Stewardship: A Rural Ghana Experience. *J. of Cultural Geography,* 24(2), 79–98. doi:10.1080/08873630709478212

Atieno V.J., Oindo B., Bosire E. (2017). Modeling Household Solid Waste Generation in Urban Estates Using SocioEconomic and Demographic Data, Kisumu City, Kenya. *Scholars J. of Arts, Humanities and Social Sciences*. 5(5):468-477. Doi: 10.21276/sjahss

Babaei A.A., Alavi N., Goudarzi G. et al. (2015). Household recycling knowledge, attitudes and practices towards solid waste management. *Resources, Conservation and Recycling*, 102, 94–100. doi:10.1016/j.resconrec.2015.06.014

Bello I.A., & Ismail M.N.B. (2016). Solid Waste Management in Africa: A Review. *International J. of Waste Resources*, *6*(2), 1. doi: 10.4172/2252-5211.1000216

Box, G. E. P., and D. R. Cox. 1964. An analysis of transformations. *Journal of the Royal Statistical Society, Series B (Methodological),* 26 (2): 211–252. https://doi.org/10.1111/j.2517-6161.1964.tb00553.x

Carus, M.; Dammer, L. (2018). The Circular Bioeconomy—Concepts, Opportunities, and Limitations. Industrial Biotechnology 14 (2): 83 – 91. http://doi.org/10.1089/ind.2018.29121.mca

Chunga R.M., Ensink J.H.J., Jenkins M.W., & Brown J. (2016). Adopt or Adapt: Sanitation Technology Choices in Urbanizing Malawi. *PLOS ONE*, *11*(8), e0161262. doi: 10.1371/journal.pone.0161262

Debreu, G. (1960). Topological methods in cardinal utility theory. In Arrow, Kenneth (ed.). Mathematical Methods in the Social Sciences,1959. Stanford: Stanford University Press. pp. 16–26. doi:10.1017/CCOL052123736X.010. ISBN 9781139052092.

de Jesus A., Mendonça S. (2018). Lost in Transition? Drivers and Barriers in the Eco-Innovation Road to the Circular Economy. *Ecological Economics, 145(), 75–89.* doi:10.1016/j.ecolecon.2017.08.001

DRC (2011). *Democratic Republic of Congo's Constitution of 2005 with Amendments through 2011*. Accessed on 21st January 2021, from: https://www.constituteproject.org/constitution/Democratic_Republic_of_the_Congo_2011.pdf?lang=en

Evans J.L., Henderson A., & Johnson N.W. (2012). Interprofessional learning enhances knowledge of roles but is less able to shift attitudes: a case study from dental education. *European J. of Dental Education,* 16(4), 239–245. doi:10.1111/j.1600-0579.2012.00749.x

El-Chichakli B., von Braun J., Lang C., Barben D., & Philp, J. (2016). Policy: Five cornerstones of a global bioeconomy. *Nature,* 535(7611), 221–223. doi:10.1038/535221a

EMF (2019). *Cities and circular economy for food.* Accessed 13th August 2020. Available from https://www.ellenmacarthurfoundation.org/assets/downloads/insight/CCEFF_Full-report_May2019_Web.pdf

FAO (2013). *Democratic Republic of Congo, Bioenergy and Food Security Projects (BEFS) Country Briefs.* FAO. Rome, Italy.

FAO (2018). *The future of food and agriculture: Alternative Pathways to 2050.* FAO. Rome, Italy.

FAO (2019). *Restoring Cassava Farming in Rwanda*. Accessed on 29th January 2021, from: http://www.fao.org/emergencies/fao-in-action/stories/stories-detail/en/c/1208321/

FAO (2020). *Democratic Republic of the Congo: Situation Report. FAO.* Rome, Italy.

FDRE (1997). *Environmental Policy of Ethiopia*. Federal Democratic Republic of Ethiopia, Addis Ababa, Ethiopia.

FDRE (2010). *Ethiopia's agricultural sector policy and investment framework (PIF) 2010-2020*. Federal Democratic Republic of Ethiopia, Addis Ababa, Ethiopia.

Ferronato N., & Torretta V. (2019). Waste Mismanagement in Developing Countries: A Review of Global Issues. *International J. of Environmental Research and Public Health*, *16*(6), 1060. doi: 10.3390/ijerph16061060

Fishburn, C. P. (1968). Utility Theory, Management Science, 14 (5), 263-382, https://doi.org/10.1287/mnsc.14.5.335

Fleischhacker, S. E., Evenson, K. R., Rodriguez, D. A., Ammerman, A. S. (2011). A systematic review of fast food access studies, Obesity Reviews, 12, (5): e460-e471, https://doi.org/10.1111/j.1467-789X.2010.00715.x

Frankema E. (2014). Africa and the Green Revolution A Global Historical Perspective. *NJAS - Wageningen J. of Life Sciences*, 70-71, 17–24. doi: 10.1016/j.njas.2014.01.003

French, S.A., Tangney, C.C., Crane, M.M. et al. (2019). Nutrition quality of food purchases varies by household income: the SHoPPER study. *BMC Public Health*, 19, 231. https://doi.org/10.1186/s12889-019-6546-2

Gatune, J., Ozor, N. and Oriama, R. (2021), "Modelling bioeconomy futures for Eastern Africa", *Foresight*, 23 (2): 226-241. https://doi.org/10.1108/FS-03-2020-0032

Geissdoerfer M., Savaget P., Bocken NMP., & Hultink E.J. (2017). The Circular Economy – A new sustainability paradigm? *J. of Cleaner Production*, 143, 757–768. doi: 10.1016/j.jclepro.2016.12.048

GGGI (2018). *Kampala Municipal Solid Waste Value Chain Mapping*. Global Green Growth Institute, Uganda, Kampala.

GGGI (2019). *Solid Waste Management in Secondary Cities of Rwanda - Muhanga & Huye: Situation assessment and potential intervention areas*. Global Green Growth Institute, Seoul, Republic of Korea.

GCT (2022). Violence in the Democratic Republic of Congo, Global Conflict Tracker. Accessed on 10th February 2022 from: https://www.cfr.org/global-conflict-tracker/conflict/violence-democratic-republic-congo

Greene, W. (2009). Discrete Choice Modelling. Palgrave Handbook of Econometrics, 473–556. doi:10.1057/9780230244405_11

Greene, H.W. (2012). Econometric Analysis. 7th Edition. Pp.801-839. Pearson, London, UK.

Hazen B.T., Mollenkopf D.A., Wang Y. (2016). Remanufacturing for the Circular Economy: An Examination of Consumer Switching Behavior. *Business Strategy and the Environment.* doi:10.1002/bse.1929

Inglehart R., Baker E.W., (2000). Modernization, Cultural Change, and the Persistence of Traditional Values. *American Sociological Review*, 65 (1), 19–51. doi:10.2307/2657288

Issa I., Delbrück S., & Hamm U. (2019). Bioeconomy from experts' perspectives – Results of a global expert survey. *PLOS ONE*, 14(5), e0215917. doi: 10.1371/journal.pone.0215917

Jara-Samaniego, J., Pérez-Murcia, D. M., Bustamante, A. M. et al. (2017). Composting as sustainable strategy for municipal solid waste management in the Chimborazo Region, Ecuador: Suitability of the obtained composts for seedling production, *Journal of Cleaner Production*, 141, 1349-1358, https://doi.org/10.1016/j.jclepro.2016.09.178.

Jurgilevich A., Birge T., Kentala-Lehtonen J. et al. (2016). Transition towards Circular Economy in the Food System. *Sustainability*, *8*(1), 69. doi: 10.3390/su8010069

Kaza S., Yao, L., Bhada-Tata P.,Van Woerden F. (2018). *What a Waste 2.0. A Global Snapshot of Solid Waste Management to 2050.* The World Bank, Washington DC – USA. DOI: 10.1596/978-1-4648-1329-0

Kershaw, H. E., Hartley, C., McLeod, C., Polson, P. (2021). The Sustainable Path to a Circular Bioeconomy, *Trends in Biotechnology*, 39, (6): 542-545, https://doi.org/10.1016/j.tibtech.2020.10.015.

Kirchherr J., Piscicelli L. (2019). Towards an Education for the Circular Economy (ECE): Five Teaching Principles and a Case Study. *Resources, Conservation and Recycling*, 150: 104406. doi:10.1016/j.resconrec.2019.104406

Kirchherr J., Piscicelli L., Bour R. et al. (2018). Barriers to the Circular Economy: Evidence from the European Union (EU). *Ecological Economics*, 150(C), 264-272. DOI: 10.1016/j.ecolecon.2018.04.028

Kirchherr J., Reike D., & Hekkert M. (2017). Conceptualizing the circular economy: An analysis of 114 definitions. R*esources, Conservation & Recycling*, 127, 221–232. doi: 10.1016/j.resconrec.2017.09.005

Korhonen J., Honkasalo A., & Seppälä J. (2017). Circular Economy: The Concept and its Limitations. *Ecological Economics*, 143, 37–46. doi: 10.1016/j.ecolecon.2017.06.041

Korhonen J., Nuur C., Feldmann A., & Birkie S.E. (2018). Circular economy as an essentially contested concept. *J. of Cleaner Production*, 175, 544–552. doi: 10.1016/j.jclepro.2017.12.111

Krütli P., Nef D., Zumwald M., Haupt M., Harlay J., Stauffacher M. [Eds.] (2018). *Waste Management in the Seychelles – Pathways for Systemic Change*. USYS TdLab Transdisciplinary Case Study 2018. ETH-Zurich, USYS TdLab. Switzerland

Kyomuhendo G.B. (2003). Low Use of Rural Maternity Services in Uganda: Impact of Women's Status, Traditional Beliefs and Limited Resources. *Reproductive Health Matters, 11(21), 16–26.* doi:10.1016/s0968-8080(03)02176-1

Liverpool-Tasie L.S.O., Omonona B.T., Sanou A., Ogunleye W.O. (2016). Is increasing inorganic fertilizer use for maize production in SSA a profitable proposition? Evidence from Nigeria. *Food Policy,* 67, 41–51. doi:10.1016/j.foodpol.2016.09.011

Long, J. S., Freese, J. (2014). Regression Models for Categorical Dependent Variables Using Stata. Third Edition. Stata Press. Texas, USA

Lwin O.M., Shin W., Yee H.Z.A., & Wardoyo J.R. (2017) A Parental Health Education Model of Children's Food Consumption: Influence on Children's Attitudes, Intention, and Consumption of Healthy and Unhealthy Foods. *J. of Health Communication*, 22:5, 403-412, DOI: 10.1080/10810730.2017.1302523

Majumdar K., Sanyal S.K., Dutta S.K. et al. (2016) *Nutrient Mining: Addressing the Challenges to Soil Resources and Food Security*. In: Singh U., Praharaj C., Singh S., Singh N. (eds) Biofortification of Food Crops. Springer, New Delhi. https://doi.org/10.1007/978-81-322-2716-8_14

Malik T., Rawat S. (2021) Microbial Valorization of Coir Pith for Development of Compost and Bioethanol Production. In: Shah S., Venkatramanan V., Prasad R. (eds) Bio-valorization of Waste. Environmental and Microbial Biotechnology. Springer, Singapore. https://doi.org/10.1007/978-981-15-9696-4_1

McCarthy, B., Liu, H.-B. and Chen, T. (2016), Innovations in the agro-food system: Adoption of certified organic food and green food by Chinese consumers, *British Food Journal*, 118 (6), 1334-1349. https://doi.org/10.1108/BFJ-10-2015-0375

Mohamad, S. S., Rusdi, S. D., Hashim, H. N. (2014). Organic Food Consumption among Urban Consumers: Preliminary Results,

Procedia - Social and Behavioral Sciences, 130, 509-514, https://doi.org/10.1016/j.sbspro.2014.04.059

Mukong, K. A., & Nanziri, E. L. (2021). Social networks and technology adoption: Evidence from mobile money in Uganda, Cogent Economics & Finance, 9:1, DOI: 10.1080/23322039.2021.1913857

Muniandy S., Kuan-Siew K., Thurasamy R., Ping A.T. (2019). The role of social media on recycling behaviour. *Sustainable Production and Consumption* 20:365-374, doi: 10.1016/j.spc.2019.08.005

Munyegera, G. K., and Matsumoto, T. (2018). ICT for financial access: Mobile money and the financial behavior of rural households in Uganda. Review of Development Economics, 22 (1): 45 – 66. https://doi.org/10.1111/rode.12327

Muscat, A., de Olde, E.M., Ripoll-Bosch, R. et al. (2021). Principles, drivers and opportunities of a circular bioeconomy. *Nature Food* 2, 561–566. https://doi.org/10.1038/s43016-021-00340-7

Nata, J.T., Mjelde, J.W. & Boadu, F.O. (2014). Household adoption of soil-improving practices and food insecurity in Ghana. Agric & Food Security, 3, 17. https://doi.org/10.1186/2048-7010-3-17

Njuki J., Kaaria S., Chamunorwa A., & Chiuri W. (2011). Linking Smallholder Farmers to Markets, Gender and Intra-Household Dynamics: Does the Choice of Commodity Matter? *The European J. of Development Research,* 23(3), 426–443. doi:10.1057/ejdr.2011.8

Pelster D., Rufino M., Rosenstock T. et al. (2017). Smallholder farms in eastern African tropical highlands have low soil greenhouse gas fluxes. *Biogeosciences,* 14(1),187–202. doi:10.5194/bg-14-187-2017

Pogash C., (2008). *Free Lunch Isn't Cool, So Some Students Go Hungry.* The New York Times, San Francisco, USA.

Purdam K., Garratt E.A., Esmail A. (2015). Hungry? Food Insecurity, Social Stigma and Embarrassment in the UK. *Sociology,* (50): 6, 1072-1088. https://doi.org/10.1177/0038038515594092

Recous S., Lashermes G., Bertrand I. et al. (2019). C–N–P Decoupling Processes Linked to Arable Cropping Management systems in relation with intensification of production. *Agroecosystem Diversity,* 35-53. doi:10.1016/B978-0-12-811050-8.00003-0

RR (2015). *Solid Organic Fertilizer – Specification*. Republic of Rwanda, Accessed 3rd July 2020. Available from: https://portal.rsb.gov.rw/catalogue.php?id=OTg3NjU0MzIxMA&keywords=fertilizer&catalogueareaid=

RR (2017). *National Agriculture Policy.* Republic of Rwanda, Kigali, Rwanda

RUNRES (2019). *The rural-urban nexus: Establishing a nutrient loop to improve city region food system resilience (Phase 1)*. https://runres.ethz.ch/approach/

Sekabira H., Nalunga, S. (2020). Farm Production Diversity: Is It Important for Dietary Diversity? Panel Data Evidence from Uganda. *Sustainability, 12(3), 1028.* doi:10.3390/su12031028

Sekabira H., & Qaim, M. (2017). Can mobile phones improve gender equality and nutrition? Panel data evidence from farm households in Uganda. *Food Policy, 73, 95–103.* doi: 10.1016/j.foodpol.2017.10.004

Sekabira, H., & Qaim, M., (2017b). Mobile Money, Agricultural Marketing, and Off-Farm Income in Uganda. Agricultural Economics, 48 (5): 597 – 611, https://doi.org/10.1111/agec.12360

Sulaiman, N.; Yeatman, H.; Russell, J.; Law, L.S. (2021). A Food Insecurity Systematic Review: Experience from Malaysia. Nutrients, 13, 945. https://doi.org/10.3390/nu13030945

Tangian, A. (2004). A model for ordinally constructing additive objective functions. *European Journal of Operational Research*. 159 (2): 476–512. doi:10.1016/S0377-2217(03)00413-2.

van der Wiel B.Z., Weijma J., van Middelaar C. et al. (2019). Restoring nutrient circularity: A review of nutrient stock and flow analyses of local agro-food-waste systems. *Resources, Conservation & Recycling: X, 3,* 100014. doi: 10.1016/j.rcrx.2019.100014

Van Ittersum M.K., van Bussel L.G.J., Wolf J. et al. (2016). Can sub-Saharan Africa feed itself? *Proceedings of the National Academy of Sciences, 113(52),* 14964–14969. doi:10.1073/pnas.1610359113

Vogel S. (2020). *The Role of Knowledge in the Acceptance of Organic and Human Waste Recycling: The Case of Rwanda's Food Systems.* Master Thesis. ETH – Zurich, Switzerland.

WFP (2010). *How to Conduct a Food Commodity Value Chain Analysis.* WFP, Rome, Italy.

Williams J. (2019). Circular Cities: Challenges to Implementing Looping Actions. *Sustainability*, *11*(2), 423. doi: 10.3390/su11020423

Wu, L., Xu, L., Zhu, D., Wang, X. (2012). Factors Affecting Consumer Willingness to Pay for Certified Traceable Food in Jiangsu Province of China, Canadian Journal of Agricultural Economics, 60, (3): 317-333, https://doi.org/10.1111/j.1744-7976.2011.01236.x

APPENDIX

Table A1: Regulatory initiatives or policies on CBE practices across countries

Country	Policy / its initiative	Source	Ministry responsible	Guidance on CBE practices (what the policy promotes)
**Rwanda**	Green Growth and Climate Resilience (GGCR)	RR, 2011 and expert interviews	Environment, and Kamonyi Municipality	Use of clean energy like methane to replace oil-powered plants, and CO_2 Forest sequestration<br>Resource recovery & reuse, agro ecology, organic composting, and wastewater irrigation, integrated soil management
	Solid organic fertilizer specifications	RR, 2015	Bureau of standards	Developed standards for use of organic fertilizers<br>Prohibits use of raw human waste (urine & feces) citing impurity risks (specification of the standard)

	National Agriculture Policy	RR, 2017	Agriculture	Use of organic fertilizers to improve soil fertility<br>Research on bio-fertilizer technologies like composting
	Strategic Plan for the Transformation of Agriculture 2018-2024	RR, 2017	Agriculture	Promote utilization of organic waste to improve and sustain soil fertility<br>Promote organic farm inputs (e.g., vermi-composting, organic fertilizers, enriched compost)
**DRC**	Constitution of the DRC	DRC, 2011, and Expert interviews	Environment and Bukavu Municipality	Management of waste (Articles 54, and 55)<br>Nature conservation
	Sur l'hygiene publique dans les agglomerations.	DRC, 1959; DRC, 2006; DRC 2015 and Expert interviews	Constitution	General regulations on handling and processing waste to protect environment and enhance public health<br>General regulations about latrines, human waste (Articles 3,4, 6 and 10)
	Portant principes fondamentaux relatifs à la protection de l'environnent.	DRC, 2011; DRC 2018	Environment	General regulations about agricultural inputs (Articles 28, and 31)
**Ethiopia**	Agricultural Sector Policy and Investment Framework (PIF)	FDRE, 2010	Agriculture and Rural Development	Use of organic fertilizers to manage soil fertility<br>Conservation of natural resources<br>Innovations that elevate soil organic carbon
	Environmental Policy of Ethiopia	FDRE, 1997, and Expert Interviews	Environment, Forestry and Climate Change	Use of organic matter, green manure, farmyard manures, and compost to improve soil structure<br>Use of agricultural residues, on-farm produced forage, and fodder as inputs for animal feeds
	Environmental Pollution Control Proclamation (No. 300)	FDRE, 2002<br><br>FDRE, 2014	Environment	General guidelines about waste disposal

Bibliography to Table A1.

DRC (1959). 74-345 Sur l'hygiene publique dans les agglomerations. Accessed 3rd September, 2019 from: http://www.leganet.cd/Legislation/Droit%20Public/SANTE/O.74.345.28.06.1959.htm

DRC (2006). Constitution de la Republique Democratique du Congo. Accessed 3rd September, 2019 from: https://www.wipo.int/edocs/lexdocs/laws/fr/cd/cd001fr.pdf

DRC (2011). Portant principes fondamentaux relatifs à la protection de l'environnent, Kinshasa, DRC.

DRC (2015). N°15/026 relative à l'eau. Accessed on 5th September, 2019 from: https://www.leganet.cd/Legislation/Droit%20economique/Eaux/Loi.15.026.31.12.2015.html#TIV

DRC (2018). N°18/035 fixant les principes fondamentaux à l'organisation de la santé publique. Accessed 19th September 2019, from: http://www.leganet.cd/Legislation/Droit%20Public/SANTE/Loi.18.035.13.12.2018.html

FDRE (2002). *Environmental Pollution Control Proclamation (No. 300).* Federal Democratic Republic of Ethiopia, Addis Ababa, Ethiopia

RR (2011). *Green Growth and Climate Resilience: National Strategy for Climate Change and Low Carbon Development.* Republic of Rwanda, Kigali, Rwanda

Figure

Click here to access/download;Figure;Figure 1.tif

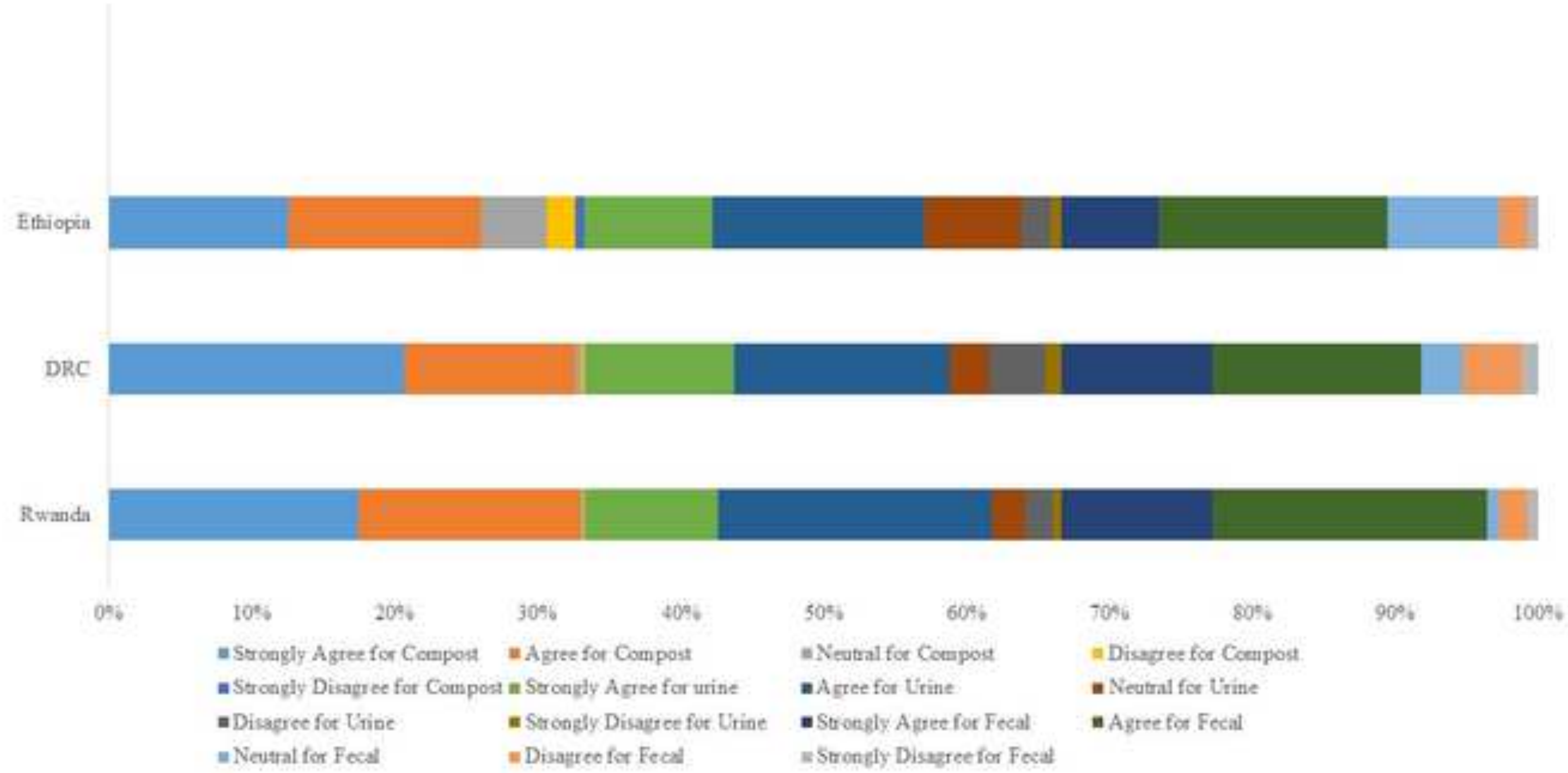

Click here to access/download
Supporting Information - Compressed/ZIP File Archive
Suplementary Material.zip

Response to Reviewers

Editor Comments:

Circular bio-economy is a "catchy" topic currently. An interesting topic alone is insufficient grounds for publication. As pointed out by some of the reviewers and my opinion as well, a very clear definition of what you consider part of the scope and what not is needed. We re-define clearly that we refer to cautious reducing, recycling, and reuse of waste using biological processes in a particular food system, where production must have occurred – otherwise such a food system would still be linear, and only leading ultimately to a circular globe, that may not be necessarily beneficial to the particular food system, where we intend to benefit consumers. We also cite more literature to justify this preposition. Lines 68 – 90 and 158 – 178

Re-use of material on-farm has been traditional practice in agriculture world-wide for ages. Circularity goes beyond that, even when dealing with transforming production systems where traditional re-use of material is under pressure. Exactly, and we make this clear, that this is not merely reuse of waste material on farm – but instead a cautious effort, that uses biological processes to reduce waste, recycle, and reuse waste effectively in particular food systems where production occurs. We also expound that such a CBE model has more than merely recycling to include objectives like reduction of fossil energy use, creation of jobs and businesses, and producing higher value products like quality compost, food, feeds, and bioenergy. Lines 158 – 178 and

A clear problem statement is needed and from there a clear definition of the materials and methods needed to address the question. We now clearly state the problem– being scanty knowledge on CBE practices which stifles proper design, development, and implementation of CBE practices in African food systems. (Lines 110 – 112). We also clearly define the research questions (Lines 139 – 145), and elaborate the analytical methodology used to analyse data and underlying assumptions to maintain the validity of the estimations. Lines 249 – 305. The statistical analysis besides not being robust, as pointed out by the reviewers is not clearly focussed on answering a research question. we make our analysis robust by defining the theory that leads us to the choice of our analytical model, and then analyse results with both ordered logistic (which we validate and realize that key underlying assumption of the proportional odds is violated), and the generalized ordered logistic regression which corrects for the violation of the proportional odds assumption. We also briefly but clearly define the proportional odds assumption for our readers. Lines 249 – 305.

Reviewers' comments:

Reviewer #1:

The paper deals with the study on „Circular Bioeconomy in African Farming Systems: What is the status quo? Insights from Rwanda, DRC, and Ethiopia". The presented topic is of high professional and practical interest bringing a significant added value to potential target group of readers. Research contribution in the manuscript can be identified so this study can be justified as innovative. The overall writing style reflects a logical clear concept. Hopefully, my remarks, observations and possible suggestions might bring the authors benefits for the enhancement of the manuscript to be published properly. Accordingly, I am stating my comments below.
Title
The title reflects the objective and content of the paper.
Abstract
The abstract provides a structured summary including contextual background, and result,

conclusion, and implications of key findings, etc. The keywords are adequate.

Introduction

This part of the manuscript is properly designed in a correct explanatory way. The most relevant part is the introduction section that gives a perfect context for the justification of the research. This section is very well based and combined with literature review part. The introduction section describes the main purpose of the study but the short explanation of research methodology, we now sufficiently expand our research methodology to nearly six pages – clearly explaining all aspects of the survey including expert interviews, and how these were handled, explaining the theory underlying our model choice for data analysis, the underlying assumptions to our model, and how we tested to validate that the assumptions were not violated, and if violated, how we corrected for the same Lines 249 – 305. key research questions these are now more clearly stated in an outline format. Lines 139 – 145. and the structure of the study are missing and this is now added, Line 152 – 154.

Methods and Data

The methodology applied seems to be unsatisfactory. The binary outcome variable is not determined. Here the reviewer had perhaps misunderstood us, and we make this clear by avoiding all binary nature of variables – which we had earlier considered at YES or NO for households whether they were aware or not, knowledgeable, or not, and supportive or not – for each country. This was simplistic and we have re-analysed data with proper ordinal scales (strongly agree, agree, neutral, disagree, and strongly disagree). Lines 249 – 305. I counted three categories anyway (aware, knowledgeable, supportive). These were variables but not categories of variables, and we have made this clearer in the revised version. Awareness and knowledgeability anyway very ambiguous concepts in this context. We use the terms based on a generalized perception of respondents – where awareness is merely having heard of such a practice, and Knowledge is having any skill about how that practice can be realized, and support is generally feeling if respondent would accept / recommend such a practice to other community members. So indeed, they are used in a broader view – which fit the purpose of our study as an exploratory one. Lines 310 – 314. I know that the model was re-estimated with a different binary dependent variable, which is even worse than the previous one. 1 means strongly agree, 0 otherwise. We abide by the guidance of the reviewer and re-estimate all models with an ordinal regression considering all outcomes' categories Lines 357 – 439. I suggest the authors to use an ordinal dependent variable with a Likert scale and use Ordinal regression (PLUM procedure to estimate this kind of regression) or at least multinomial logistic regression. We now both the ordered logistic regression and the generalized ordered logistic regression to estimate the model Lines 357 – 439. However, this is not the only conceptual and methodological mistake in the manuscript. Actually, there is no report on the model fit (Nagelkerke R squared, etc…). The model fits for the interpretated generalized ordered logit model are well presented in the revised version. Lines 357 – 439. Furthermore, there is no investigation on the assumption/conditions, we investigate the assumption of proportionality of odds for the ordered logit, which our data fails and then we ran the generalized ordered logit model to correct for the violation of the assumption, the GOL also controls for multi-collinearity. Lines 357 – 439. which should be satisfied by the application of logistic regression (multicollinearity for example). Model validation with cross-validation techniques is missing as well. We present Chi2 values and Pseudo R2 for validation of the model Lines 357 – 439. It is questionable that parameters could be significant (Table 6: for example -0.003) due to the very small standard deviation. Certain coefficients being small is not necessary a models problem but a challenge with the units used – for instance for distance we used kilometres which are a more conservative scale, than meters, hence forth we try to alleviate this problem by clearly indicating the units used for each variable, and as well in the discussions translating these differences in percentage points which are much easier to understand for readers. There is no information about the outliers and missing values. This we had mistakenly left out – but we mention now that extreme outliers were followed up with the respondents and corrected, for instance two household head in DRC had age over 121 years, and since we had

contacts, we followed up. Otherwise, for the normal outliers we used the box-cox transformations, to generate normalized variables that wouldn't change scale in interpretations. Lines 300 – 305. I have no idea about the software used to fit this model. We now mention this in the paper, STATA/SE 16.0 – but we had mistakenly left it out. Lines 214. To sum up the manuscript does not seem to be very original. We re-write the manuscript better to give it an excellent original nature that it is.
Results
The manuscript reports the results grounded in a statistical sense, however, comparing and combining results with previous studies are missing This we now do – showing clearly what previous studies yielded results that were similar. 487 – 609. The regression results are questionable (see my comments ont he methodology).

Analysis and Discussion
The discussion part including limitations of the study is well written.
Conclusion
Conclusions reflect all main findings appropriately. In this section the main ideas of the manuscript are presented, the obtained results and their novelty are demonstrated. Nevertheless, the value added of this part is limited by the insufficient methodology used for the analysis. analytical methodology is more clearly presented and explained Lines 357 – 439.

Addressing these concerns would entail major revisions to this manuscript in order to be considered for publication. On the basis of these observations, this paper is recommended for publication after major revisions detailed in my comments are satisfactorily completed.

Reviewer #2: The paper addresses the status quo of the circular bioeconomy (CBE) in three African countries. The authors use a survey among farmers and consumers to explore "from key food value chains (cassava, coffee, and banana)" the "existing CBE practices; awareness, knowledge, and support for CBE practices; consumers' opinions on eating foods grown on CBE fertilizers (CBE foods), and determinants for such opinions." The authors use the results of the survey for a descriptive analysis and a logistic regression analysis for assessing the status. The authors draw conclusions based on the results with respect to the status of the CBE in the study region and policy support for stimulating the CBE.
The paper has a number of short-comings. The theoretical s not well explained We now explain the underlying theory of our empirical estimations. Lines 248 – 262. The link with the empirical study is not well established and excellently link the theory to empirics. 263 – 274. The empirical study seems to be ad-hoc. Some elaborations, had been left-out for fear of making the paper bulky – currently about 12,000 words – nevertheless, key explanations in data collected Lines 180 – 206, theory underlying our analysis Lines 248 - 262, elaborated problem lines 110 – 112, and research questions lines 139 – 145, validation of empirical model etc. are now all done lines 271 – 274. The ad-hoc empirical study has a number of short-comings, some are listed below, that undermine the conclusions being drawn.
The authors use the concept of the CBE. This concept is not well introduced. Again, for fear of going deep into the conceptual definitions of CBE, we avoided detailed background earlier – but now we provide a helpful but concise detail to help readers clearly understand how we come up with the CBE concept, which comes from two other concepts Circular economy and bioeconomy, lines68 – 79, and 157 – 177. Several authors have pointed out that planet earth is a closed system. This we and we have elaborated in the paper, but our focus is the circular/closed particular food system – which is key to sustainable food production, otherwise, if food waste is generated in one city food system after production, and waste is dumped in rivers where waste is decomposed but nutrients washed away to river banks where food production isn't happening – such a food system is still linear, even though in general the globe will be closed/circular. We make this distinction in the revised manuscript. Lines 50 – 62. The material flow on plant earth is circular. The difference

between the linear and the circular system the authors refer to in their study needs to be more clearly pointed out and in particular the implications for the nutrient cycle. This has clearly been done as stated in response above, lines 170 – 177. Why does not using organic waste breaks the nutrient cycle? What specific cycle do the authors refer to? This has been well articulated in the introduction and methods section – specially under the circular bioeconomy concept heading. Lines 157 – 177

The model the authors presents: "Under this model, production would still mostly occur in rural areas, and consumption in town centres. However, after urban consumption, organic waste (food, household, and human) would be collected, recycled, and returned to rural areas for reuse on farms." We make it clear that this is the CBE model – not the CE model through the paper.

In the study the complexity of this process has not been considered. The survey only includes consumers and farmers, while the other actors of the value are not included. We elaborate the complexity of the process that indeed CBE practices need resources like time, money, and labour – and that some actors in certain value chains couldn't be traced under the random sampling mechanism but that we employed a snowball one as well. Other actors, had not been presented to minimize space and as well because their numbers were not enough for statistical analysis across all countries, then we had left them out. We now included them but explain why still we exclude some from the analysis – because in some value chains, some segments were completely unavailable. Lines 180 – 241. The process of collecting, recycling, and reusing is not a costless process. This also requires resources. The process might be too expensive and it might be cheaper to not use recycled organic material according to the model presented or to use other organic material. This aspect has not been discussed nor considered. This has been made very clear in the discussions of our results, that indeed the CBE process involves costs, and that food processors were selling organic waste for money, but we didn't analyze the economic viability of CBE practices in this paper. Lines 516 – 539

What is worrying is that the use of human waste as compost is seen as an important part of the CBE. Applying human waste as compost is forbidden in many countries and rightly so for food safety reasons. We show clearly in our discussions of reviewed policies that indeed human waste is prohibited in some countries like DRC, but also report our field experiences that it was being treated and used in Rwanda from old pit-latrines. However, we acknowledge safety and health risks around the practice which could indeed be rightly keeping people off using it especially the educated ones, as our results show. We highlight that there is need to certify the safety of the product first, Lines 227 – 241. (Some countries allow treated human waste to be used, but often only for non-food crops). The authors seem not be aware about the food safety dangers related to the use of human waste. This we have elaborated clearly and the need for certification, as the process has been kick-started in Rwanda, lines 502 – 505. Some of the respondents might be aware about these problems and this may have an impact on the survey responses. In the survey the question is related to treated human waste. Yes – but this wasn't the more complex treatment that CBE innovations would later prefer to install. This was merely keeping the waste anaerobically for some time to kill pathogens. We now clearly state this in the paper, lines 494 – 495. Treatment of human waste is not simple and by no means costless. This issue has not been addressed in the paper (and not considered in the empirical analysis). We now clearly clarify on the issue of costs – and even refer to supporting literature on costs around composting, lines 524 – 530.

The empirical analysis uses a logistic regression to estimate household attributes on CBE awareness, knowledge, and support. The regressions are run by country. This is appropriate as the cases and countries are different. The results cannot be compared without taking the differences into account. We now consider country differences by running a pooled generalized ordered logistic regression but control for country specific effects, which still gives us the different effects of belonging to the different countries – just as we would do running models per country. However, this time we can as well compare results across countries. Lines 356 – 450. This undermines the policy conclusions, which are already limited as the costs related to recycling organic waste have not been taken into consideration. Our policy conclusions are now inclusive of country effects – thus can be more

externally valid. We also do not talk about costs in our conclusions but we make it clear that a CBE innovation being cost-effective and commercially viable is what will attract community and government support. Hence, indirectly we make reference to costs. Lines 639 – but also in genera; revised manuscript.

Reviewer #3: Data collection

Only farmers and consumers were interviewed yet the value chains have many actors and sometimes the biggest components of waste is generated in between e.g. in logistics and processing parts of the value chain We had this data before, but for fear of bulking the paper, we had left it out – now we present it and make it clear what we do with which data and why, and what data we use for the analysis done in this paper. Lines 180 – 226.
Also rather than just search for keywords on internet to find whether there is policy on use waste, yet most government websites have little data and are not frequently updated. it would have been better to just interview officers in the relevant government ministries/. Also much of the waste in a city is handled by cities. The officers in the cities are not interviewed either Indeed, municipal authorities specifically the mayors and their support units like that on environment and waste management, as well as experts in ministries had been interviewed after an online search on CBE related policies had been done for each country. We now elaborate this process more clearly and as well present an annex of the key outputs of the policy reviews – in Table A1. Lines 227 – 241.

Garbage collection in cities also has private sector actors. Indeed garbage collection is one of the fist sector to see public-private partnerships. This was captured in the component of processors in food value chains – because in most municipalities both households and processors had been directed by policy to get attached to garbage collection companies. In fact key project partners in Rwanda and DRC, are garbage collectors – and we now clarify this in the paper. Lines 183 – 185.

While these actors need not have been surveyed, key informants interviews would have given a deeper insight in the CBE value chain. Key informant interviews had been done with our local partners, municipal authorities, and ministerial staff, and we now clearly mention this. However, these interviews led more to the justification of data which we analysed for the technical report, and partly used here. We clearly elaborate this was done in the revised manuscript. Lines 230 – 235.

Regression Model

Why not use commodity dummies and also country dummies to capture the separate effecs of commodity chain and also country effect, A pooled regression can give a much richer results and easier to interpret.

We re-estimate all results with a pooled generalized ordered logit regression and even control for country/commodity effects. Country effects, automatically control for other and commodity effects because each commodity was grown in a different country. Lines 356 – 443.
Usually we test the model for fitness before we do regression e.g check for multi-collinearity etc. No check seems to have been done This time we do the tests. First, we ran the ordered logit regression and test for the proportionality of odds assumption across all response categories of outcome variables, where the resultant test Chi2 value was significant in all regressions thus violating this assumption. Hence, to correct for this violation, we ran the generalized ordered logit (GOL) regression which controls also for collinearity among variables and drops variables that present collinearity. Model fit estimates for the GOL are presented below the tables of results, including the Pseudo R2, which is between 6 to 40%, an acceptable range for especially survey data. The models Chi2 value is also presented and is significant in all models. Lines 356 – 443.

Some explanations are weak

Line 76-79

Increased crop or animal productivity from use of recycled nutrients (asssuming low production costs of recycled inputs, since organic waste is abundantly available) could also increase household incomes via improved crop and animal yields, or reduce household expenditure via 9 lower food cost caused by greater food supply

Transport cost cane be quite high sometime 50% of the cost depending on the value chain. Likewise transporting organic waste maybe quite expensive as logistics of collecting and transporting maybe even more. May require specialized vehicles and in cities only old trucks seem to do this job. This consideration is taken care of, and the explanation is made stronger. We highlight that our assumption of low costs can prevail if the recycling process is efficient as the CBE model advocates – efficiency we elaborate that can be achieved if processing is done on dumpsites, and homes without having to transport the bulky waste. Also, sorting waste at source before being taken at dumpsites can help reduce sorting costs. Lines 95 – 96

Line 363-365

In that sense, it is also significant that we found dependence on 364 agriculture to be associated with supporting CBE practices. This is most likely because agriculture 365 forces farmers to practice CBE, probably due to a lack of other alternatives.

Could it be that farmers with high dependence on agriculture are also more knowledgeable about benefits of CBE rqther than adopting due to lack of alternatives? This suggestion is appreciated and considered in the revised manuscript. Lines 508 – 509

Line 373-376

Severe food insecurity was associated with less awareness, knowledge, and support for 374 CBE practices (Table 5). Food access is a basic essential human need, without which human 375 faculties are impaired, for instance proper growth, and understanding, (Spencer 2008). Therefore, 376 CBE innovations could need to prioritize household food security to succeed.

Very odd explanation. That lowered IQ of food insecure household has impacted their understanding of CBE. This is highly speculative and such conclusion can be made with very careful study. It could be something that is driving the food insecurity could also be driving their lack of knowledge. For example conflict can drive both and this is a much simpler explanation. We appreciate this suggestion and incorporate the same in the new manuscript. Lines 551 – 553

Line 386-393

he inverse relationship between household distance to town and diminishing likelihood 387 of acceptance to eat CBE grown food implies that urban consumers would be the more likely 388 market for CBE foods. This is most likely because rural areas are largely inhabited by communities 389 strong in cultural and traditional beliefs (Kyomuhendo 2003; Appiah-Opoku 2007). Rural 390 communities may also be less exposed to daily food insecurity challenges, as these may have more 391 land resources to grow foods, (Njuki et al. 2011). Moreover, urban consumers are less

concerned 392 about production methods of especially locally (perceived to meet organic and social values) 393 produced foods (Hempel & Hamm, 2016)

Careful with this conclusion. Urban residents are becoming increasingly interested. Food safety sustainability is now key concerns. Traceability is being demanded. Though this is pressure is yet to become widespread in Africa. You cannot divorce promotion of CBE from sustainability demands. This suggestion is much appreciated and incorporated – we totally drop the explanation of being less concerned but strengthen the increasing reality of urban consumers demanding for safety certified foods. Lines 542 – 544

Line 401-403

Thus, the role of 402 government, next to the private sector's role for valorisation of waste, seems to be crucial for the 403 establishment of a CBE
This could have been explored as part of the study. Like chemical fertilizer, inorganic fertilizers can also be commercialized by the private sector. This suggestion is appreciated and finally totally dropped in the revised manuscript.

Line 406-407

household food insecurity challenges due to having more persons to feed (Kaza et al. 2018). Hence, 407 resorting to resource conservative and low cost produced foods, such as CBE foods, is logical. The assumption that CBE foods are cheaper needed to be supported. Indeed, the research should have tried to understand this. This is indeed a critical question that is key to uptake of CBE and we cannot make this assertion without any supporting evidence. We re explain this better with supporting evidence. Lines 574 – 579

Line 419-427

Finally, in DRC, households with severe food insecurity seem to be more likely to eat CBE 420 foods grown using compost, but severely food insecure households in Rwanda and Ethiopia seem 421 less likely to accept eat CBE foods. Interestingly, the effects are not that significant. However, this 422 may be due to some social-cultural values, yet these need longer time to change (Inglehart & Baker, 423 2000). Moreover, in some instances, social stigma has been found to negatively influence food 424 acceptance by food insecure persons/households (Pogash, 2008; Purdam et al. 2015). Therefore, 425 CBE innovations must be aware of potential social dislikes, and strengthen awareness campaigns 426 on safety, and appropriateness of CBE foods. Wrongful consumer perceptions may generate social 427 stigma against CBE foods
It makes no sense to say that the results were not that significant and then go ahead to explain the results and offer potential explanation. Results are not significant means not effect. Any explanation is of no use. Or do you mean the effects are significant but very small. This guidance is well appreciated – we abandon the explanation and explain only significant variables. Lines 547 – 568.

Line 437-440

We recommend that experimental or panel data could be used in the future to support this initial 438 research. That said, we are confident that our results reflect the CBE status quo in the studied city 439 regions and can reliably inform successful implementation and scaling of CBE innovations in those 440 regions
Given that this was an exploratory study , the outhors this should develop a section to explore some

hypothesis for other to test in the future. This can be developed from the various conclusion you have made i.e.
i. CBE innovations most likely need to prioritize knowledge and learning components in 363 setting up CBE innovations.
ii. Thus, CBE innovations could target agricultural households for demand for compost and 371 animal feeds
iii. Therefore, 376 CBE innovations could need to prioritize household food security to succeed
iv. hus, CBE 384 innovations should prioritize commercial viability of CBE products to attract male headed 385 (majority) households to agriculture based on CBE practices.
v. Thus, the role of 402 government, next to the private sector's role for valorisation of waste, seems to be crucial for the 403 establishment of a CBE
vi. Thus, CBE innovations could target households with heads of average old 412 working age, as these could even be more likely to accommodate behavioural changes. With better 413 education, understanding of concepts like CBE becomes possible (Evans et al. 2012) a
vii. Therefore, CBE innovations need to incorporate mechanisms that 418 ensure products' safety and quality
viii. Therefore, 425 CBE innovations must be aware of potential social dislikes, and strengthen awareness campaigns 426 on safety, and appropriateness of CBE foods. Wrongful consumer perceptions may generate social 427 stigma against CBE foods. This suggestion is much appreciated and incorporated in lines 622 – 640.

Line 446 and line 448-450

We reviewed country policies on CBE practices
Furthermore, policies concerning circular bioeconomy 449 appear to be insufficient, and thus need immediate attention for formulation, clarity, and 450 enforcemen

What was done does not meet the criteria of a review. This was a quick and dirty approach. Need to interview a few actors in policy making and also NGOs and also review grey literature to better understand the policies. We elaborate that we had originally done expert and key informant interviews in ministries, and municipalities, and project partners, and as well we reviewed grey literature in government reports. Lines 227 – 241.
A study done by Bio innovate on East Africa bioeconomy strategy that included Rwanda and Uganda can give some insights
General insights. Much appreciated for the insights from this study – especially in phrasing what I had done, only that I found this more applicable for a reference: *Gatune, J., Ozor, N. and Oriama, R. (2021), "Modelling bioeconomy futures for Eastern Africa", Foresight, 23 (2): 226-241.* https://doi.org/10.1108/FS-03-2020-0032. Lines 112, 127, and 530

It would also have been good to reflect on what your results mean in terms of market for CBEs and implication for value chain development. If demand is there in urban areas should we target a certain segment. For example organic foods tend to target higher income. Much appreciated suggestion and incorporated in manuscript. Lines 653 – 654.

Also given the need to have waste re-used at rural level how should value chain be re-organized to help lower the cost of taking waste back to rural areas?. This is much appreciated and incorporated in lines 659 – 661.
