# Supplementary material for: Circular bioeconomy in African food systems: What is the status quo? Insights from Rwanda, DRC, and Ethiopia
Source: PLoS One. 2022 Oct 20;17(10):e0276319. doi: 10.1371/journal.pone.0276319 (PMC9584527; doi:10.1371/journal.pone.0276319)
Supplement: Attachment — Submitted filename: RESPONSE TO REVIEWERS.docx [file pone.0276319.s004.docx]

**Response to Reviewers**

**Editor Comments:**

Circular bio-economy is a "catchy" topic currently. An interesting topic alone is insufficient grounds for publication. As pointed out by some of the reviewers and my opinion as well, a very clear definition of what you consider part of the scope and what not is needed. We re-define clearly that we refer to cautious reducing, recycling, and reuse of waste using biological processes in a particular food system, where production must have occurred – otherwise such a food system would still be linear, and only leading ultimately to a circular globe, that may not be necessarily beneficial to the particular food system, where we intend to benefit consumers. We also cite more literature to justify this preposition. Lines 68 – 90 and 158 – 178

Re-use of material on-farm has been traditional practice in agriculture world-wide for ages. Circularity goes beyond that, even when dealing with transforming production systems where traditional re-use of material is under pressure. Exactly, and we make this clear, that this is not merely reuse of waste material on farm – but instead a cautious effort, that uses biological processes to reduce waste, recycle, and reuse waste effectively in particular food systems where production occurs. We also expound that such a CBE model has more than merely recycling to include objectives like reduction of fossil energy use, creation of jobs and businesses, and producing higher value products like quality compost, food, feeds, and bioenergy. Lines 158 – 178 and

A clear problem statement is needed and from there a clear definition of the materials and methods needed to address the question. We now clearly state the problem– being scanty knowledge on CBE practices which stifles proper design, development, and implementation of CBE practices in African food systems. (Lines 110 – 112). We also clearly define the research questions (Lines 139 – 145), and elaborate the analytical methodology used to analyse data and underlying assumptions to maintain the validity of the estimations. Lines 249 – 305. The statistical analysis besides not being robust, as pointed out by the reviewers is not clearly focussed on answering a research question. we make our analysis robust by defining the theory that leads us to the choice of our analytical model, and then analyse results with both ordered logistic (which we validate and realize that key underlying assumption of the proportional odds is violated), and the generalized ordered logistic regression which corrects for the violation of the proportional odds assumption. We also briefly but clearly define the proportional odds assumption for our readers. Lines 249 – 305.


**Reviewers' comments:**

**Reviewer #1:** The paper deals with the study on „Circular Bioeconomy in African Farming Systems: What is the status quo? Insights from Rwanda, DRC, and Ethiopia”. The presented topic is of high professional and practical interest bringing a significant added value to potential target group of readers. Research contribution in the manuscript can be identified so this study can be justified as innovative. The overall writing style reflects a logical clear concept. Hopefully, my remarks, observations and possible suggestions might bring the authors benefits for the enhancement of the manuscript to be published properly. Accordingly, I am stating my comments below.
Title
The title reflects the objective and content of the paper.
Abstract
The abstract provides a structured summary including contextual background, and result, conclusion, and implications of key findings, etc. The keywords are adequate.
Introduction
This part of the manuscript is properly designed in a correct explanatory way. The most relevant part is the introduction section that gives a perfect context for the justification of the research. This section is very well based and combined with literature review part. The introduction section describes the main purpose of the study but the short explanation of research methodology, we now sufficiently expand our research methodology to nearly six pages – clearly explaining all aspects of the survey including expert interviews, and how these were handled, explaining the theory underlying our model choice for data analysis, the underlying assumptions to our model, and how we tested to validate that the assumptions were not violated, and if violated, how we corrected for the same Lines 249 – 305. key research questions these are now more clearly stated in an outline format. Lines 139 – 145. and the structure of the study are missing and this is now added, Line 152 – 154.
Methods and Data
The methodology applied seems to be unsatisfactory. The binary outcome variable is not determined. Here the reviewer had perhaps misunderstood us, and we make this clear by avoiding all binary nature of variables – which we had earlier considered at YES or NO for households whether they were aware or not, knowledgeable, or not, and supportive or not – for each country. This was simplistic and we have re-analysed data with proper ordinal scales (strongly agree, agree, neutral, disagree, and strongly disagree). Lines 249 – 305. I counted three categories anyway (aware, knowledgeable, supportive). These were variables but not categories of variables, and we have made this clearer in the revised version. Awareness and knowledgeability anyway very ambiguous concepts in this context. We use the terms based on a generalized perception of respondents – where awareness is merely having heard of such a practice, and Knowledge is having any skill about how that practice can be realized, and support is generally feeling if respondent would accept / recommend such a practice to other community members. So indeed, they are used in a broader view – which fit the purpose of our study as an exploratory one. Lines 310 – 314. I know that the model was re-estimated with a different binary dependent variable, which is even worse than the previous one. 1 means strongly agree, 0 otherwise. We abide by the guidance of the reviewer and re-estimate all models with an ordinal regression considering all outcomes’ categories Lines 357 – 439. I suggest the authors to use an ordinal dependent variable with a Likert scale and use Ordinal regression (PLUM procedure to estimate this kind of regression) or at least multinomial logistic regression. We now both the ordered logistic regression and the generalized ordered logistic regression to estimate the model Lines 357 – 439. However, this is not the only conceptual and methodological mistake in the manuscript. Actually, there is no report on the model fit (Nagelkerke R squared, etc…). The model fits for the interpretated generalized ordered logit model are well presented in the revised version. Lines 357 – 439. Furthermore, there is no investigation on the assumption/conditions, we investigate the assumption of proportionality of odds for the ordered logit, which our data fails and then we ran the generalized ordered logit model to correct for the violation of the assumption, the GOL also controls for multi-collinearity. Lines 357 – 439. which should be satisfied by the application of logistic regression (multicollinearity for example). Model validation with cross-validation techniques is missing as well. We present Chi2 values and Pseudo R2 for validation of the model Lines 357 – 439. It is questionable that parameters could be significant (Table 6: for example -0.003) due to the very small standard deviation. Certain coefficients being small is not necessary a models problem but a challenge with the units used – for instance for distance we used kilometres which are a more conservative scale, than meters, hence forth we try to alleviate this problem by clearly indicating the units used for each variable, and as well in the discussions translating these differences in percentage points which are much easier to understand for readers. There is no information about the outliers and missing values. This we had mistakenly left out – but we mention now that extreme outliers were followed up with the respondents and corrected, for instance two household head in DRC had age over 121 years, and since we had contacts, we followed up. Otherwise, for the normal outliers we used the box-cox transformations, to generate normalized variables that wouldn’t change scale in interpretations. Lines 300 – 305. I have no idea about the software used to fit this model. We now mention this in the paper, STATA/SE 16.0 – but we had mistakenly left it out. Lines 214. To sum up the manuscript does not seem to be very original. We re-write the manuscript better to give it an excellent original nature that it is.
Results
The manuscript reports the results grounded in a statistical sense, however, comparing and combining results with previous studies are missing This we now do – showing clearly what previous studies yielded results that were similar. 487 – 609. The regression results are questionable (see my comments ont he methodology).

Analysis and Discussion
The discussion part including limitations of the study is well written.
Conclusion
Conclusions reflect all main findings appropriately. In this section the main ideas of the manuscript are presented, the obtained results and their novelty are demonstrated. Nevertheless, the value added of this part is limited by the insufficient methodology used for the analysis. analytical methodology is more clearly presented and explained Lines 357 – 439.

Addressing these concerns would entail major revisions to this manuscript in order to be considered for publication. On the basis of these observations, this paper is recommended for publication after major revisions detailed in my comments are satisfactorily completed.

**Reviewer #2:** The paper addresses the status quo of the circular bioeconomy (CBE) in three African countries. The authors use a survey among farmers and consumers to explore "from key food value chains (cassava, coffee, and banana)" the "existing CBE practices; awareness, knowledge, and support for CBE practices; consumers’ opinions on eating foods grown on CBE fertilizers (CBE foods), and determinants for such opinions." The authors use the results of the survey for a descriptive analysis and a logistic regression analysis for assessing the status. The authors draw conclusions based on the results with respect to the status of the CBE in the study region and policy support for stimulating the CBE.
The paper has a number of short-comings. The theoretical s not well explained We now explain the underlying theory of our empirical estimations. Lines 248 – 262. The link with the empirical study is not well established and excellently link the theory to empirics. 263 – 274. The empirical study seems to be ad-hoc. Some elaborations, had been left-out for fear of making the paper bulky – currently about 12,000 words – nevertheless, key explanations in data collected Lines 180 – 206, theory underlying our analysis Lines 248 - 262, elaborated problem lines 110 – 112, and research questions lines 139 – 145, validation of empirical model etc. are now all done lines 271 – 274. The ad-hoc empirical study has a number of short-comings, some are listed below, that undermine the conclusions being drawn.
The authors use the concept of the CBE. This concept is not well introduced. Again, for fear of going deep into the conceptual definitions of CBE, we avoided detailed background earlier – but now we provide a helpful but concise detail to help readers clearly understand how we come up with the CBE concept, which comes from two other concepts Circular economy and bioeconomy, lines68 – 79, and 157 – 177. Several authors have pointed out that planet earth is a closed system. This we and we have elaborated in the paper, but our focus is the circular/closed particular food system – which is key to sustainable food production, otherwise, if food waste is generated in one city food system after production, and waste is dumped in rivers where waste is decomposed but nutrients washed away to river banks where food production isn’t happening – such a food system is still linear, even though in general the globe will be closed/circular. We make this distinction in the revised manuscript. Lines 50 – 62. The material flow on plant earth is circular. The difference between the linear and the circular system the authors refer to in their study needs to be more clearly pointed out and in particular the implications for the nutrient cycle. This has clearly been done as stated in response above, lines 170 – 177. Why does not using organic waste breaks the nutrient cycle? What specific cycle do the authors refer to? This has been well articulated in the introduction and methods section – specially under the circular bioeconomy concept heading. Lines 157 – 177
The model the authors presents: "Under this model, production would still mostly occur in rural areas, and consumption in town centres. However, after urban consumption, organic waste (food, household, and human) would be collected, recycled, and returned to rural areas for reuse on farms." We make it clear that this is the CBE model – not the CE model through the paper.
In the study the complexity of this process has not been considered. The survey only includes consumers and farmers, while the other actors of the value are not included. We elaborate the complexity of the process that indeed CBE practices need resources like time, money, and labour – and that some actors in certain value chains couldn’t be traced under the random sampling mechanism but that we employed a snowball one as well. Other actors, had not been presented to minimize space and as well because their numbers were not enough for statistical analysis across all countries, then we had left them out. We now included them but explain why still we exclude some from the analysis – because in some value chains, some segments were completely unavailable. Lines 180 – 241. The process of collecting, recycling, and reusing is not a costless process. This also requires resources. The process might be too expensive and it might be cheaper to not use recycled organic material according to the model presented or to use other organic material. This aspect has not been discussed nor considered. This has been made very clear in the discussions of our results, that indeed the CBE process involves costs, and that food processors were selling organic waste for money, but we didn’t analyze the economic viability of CBE practices in this paper. Lines 516 – 539
What is worrying is that the use of human waste as compost is seen as an important part of the CBE. Applying human waste as compost is forbidden in many countries and rightly so for food safety reasons. We show clearly in our discussions of reviewed policies that indeed human waste is prohibited in some countries like DRC, but also report our field experiences that it was being treated and used in Rwanda from old pit-latrines. However, we acknowledge safety and health risks around the practice which could indeed be rightly keeping people off using it especially the educated ones, as our results show. We highlight that there is need to certify the safety of the product first, Lines 227 – 241. (Some countries allow treated human waste to be used, but often only for non-food crops). The authors seem not be aware about the food safety dangers related to the use of human waste. This we have elaborated clearly and the need for certification, as the process has been kick-started in Rwanda, lines 502 – 505. Some of the respondents might be aware about these problems and this may have an impact on the survey responses. In the survey the question is related to treated human waste. Yes – but this wasn’t the more complex treatment that CBE innovations would later prefer to install. This was merely keeping the waste anaerobically for some time to kill pathogens. We now clearly state this in the paper, lines 494 – 495. Treatment of human waste is not simple and by no means costless. This issue has not been addressed in the paper (and not considered in the empirical analysis). We now clearly clarify on the issue of costs – and even refer to supporting literature on costs around composting, lines 524 – 530.
The empirical analysis uses a logistic regression to estimate household attributes on CBE awareness, knowledge, and support. The regressions are run by country. This is appropriate as the cases and countries are different. The results cannot be compared without taking the differences into account. We now consider country differences by running a pooled generalized ordered logistic regression but control for country specific effects, which still gives us the different effects of belonging to the different countries – just as we would do running models per country. However, this time we can as well compare results across countries. Lines 356 – 450. This undermines the policy conclusions, which are already limited as the costs related to recycling organic waste have not been taken into consideration. Our policy conclusions are now inclusive of country effects – thus can be more externally valid. We also do not talk about costs in our conclusions but we make it clear that a CBE innovation being cost-effective and commercially viable is what will attract community and government support. Hence, indirectly we make reference to costs. Lines 639 – but also in genera; revised manuscript.

**Reviewer #3:** Data collection

Only farmers and consumers were interviewed yet the value chains have many actors and sometimes the biggest components of waste is generated in between e.g. in logistics and processing parts of the value chain We had this data before, but for fear of bulking the paper, we had left it out – now we present it and make it clear what we do with which data and why, and what data we use for the analysis done in this paper. Lines 180 – 226.
Also rather than just search for keywords on internet to find whether there is policy on use waste, yet most government websites have little data and are not frequently updated. it would have been better to just interview officers in the relevant government ministries/. Also much of the waste in a city is handled by cities. The officers in the cities are not interviewed either Indeed, municipal authorities specifically the mayors and their support units like that on environment and waste management, as well as experts in ministries had been interviewed after an online search on CBE related policies had been done for each country. We now elaborate this process more clearly and as well present an annex of the key outputs of the policy reviews – in Table A1. Lines 227 – 241.

Garbage collection in cities also has private sector actors. Indeed garbage collection is one of the fist sector to see public-private partnerships. This was captured in the component of processors in food value chains – because in most municipalities both households and processors had been directed by policy to get attached to garbage collection companies. In fact key project partners in Rwanda and DRC, are garbage collectors – and we now clarify this in the paper. Lines 183 – 185.

While these actors need not have been surveyed, key informants interviews would have given a deeper insight in the CBE value chain. Key informant interviews had been done with our local partners, municipal authorities, and ministerial staff, and we now clearly mention this. However, these interviews led more to the justification of data which we analysed for the technical report, and partly used here. We clearly elaborate this was done in the revised manuscript. Lines 230 – 235.

Regression Model

Why not use commodity dummies and also country dummies to capture the separate effecs of commodity chain and also country effect, A pooled regression can give a much richer results and easier to interpret.

We re-estimate all results with a pooled generalized ordered logit regression and even control for country/commodity effects. Country effects, automatically control for other and commodity effects because each commodity was grown in a different country. Lines 356 – 443.
Usually we test the model for fitness before we do regression e.g check for multi-collinearity etc. No check seems to have been done This time we do the tests. First, we ran the ordered logit regression and test for the proportionality of odds assumption across all response categories of outcome variables, where the resultant test Chi2 value was significant in all regressions thus violating this assumption. Hence, to correct for this violation, we ran the generalized ordered logit (GOL) regression which controls also for collinearity among variables and drops variables that present collinearity. Model fit estimates for the GOL are presented below the tables of results, including the Pseudo R2, which is between 6 to 40%, an acceptable range for especially survey data. The models Chi2 value is also presented and is significant in all models. Lines 356 – 443.

**Some explanations are weak**

Line 76-79

Increased crop or animal productivity from use of recycled nutrients (asssuming low production costs of recycled inputs, since organic waste is abundantly available) could also increase household incomes via improved crop and animal yields, or reduce household expenditure via 9 lower food cost caused by greater food supply

Transport cost cane be quite high sometime 50% of the cost depending on the value chain. Likewise transporting organic waste maybe quite expensive as logistics of collecting and transporting maybe even more. May require specialized vehicles and in cities only old trucks seem to do this job. This consideration is taken care of, and the explanation is made stronger. We highlight that our assumption of low costs can prevail if the recycling process is efficient as the CBE model advocates – efficiency we elaborate that can be achieved if processing is done on dumpsites, and homes without having to transport the bulky waste. Also, sorting waste at source before being taken at dumpsites can help reduce sorting costs. Lines 95 – 96

Line 363-365

In that sense, it is also significant that we found dependence on 364 agriculture to be associated with supporting CBE practices. This is most likely because agriculture 365 forces farmers to practice CBE, probably due to a lack of other alternatives.

Could it be that farmers with high dependence on agriculture are also more knowledgeable about benefits of CBE rqther than adopting due to lack of alternatives? This suggestion is appreciated and considered in the revised manuscript. Lines 508 – 509

Line 373-376

Severe food insecurity was associated with less awareness, knowledge, and support for 374 CBE practices (Table 5). Food access is a basic essential human need, without which human 375 faculties are impaired, for instance proper growth, and understanding, (Spencer 2008). Therefore, 376 CBE innovations could need to prioritize household food security to succeed.

Very odd explanation. That lowered IQ of food insecure household has impacted their understanding of CBE. This is highly speculative and such conclusion can be made with very careful study. It could be something that is driving the food insecurity could also be driving their lack of knowledge. For example conflict can drive both and this is a much simpler explanation. We appreciate this suggestion and incorporate the same in the new manuscript. Lines 551 – 553

Line 386-393

he inverse relationship between household distance to town and diminishing likelihood 387 of acceptance to eat CBE grown food implies that urban consumers would be the more likely 388 market for CBE foods. This is most likely because rural areas are largely inhabited by communities 389 strong in cultural and traditional beliefs (Kyomuhendo 2003; Appiah-Opoku 2007). Rural 390 communities may also be less exposed to daily food insecurity challenges, as these may have more 391 land resources to grow foods, (Njuki et al. 2011). Moreover, urban consumers are less concerned 392 about production methods of especially locally (perceived to meet organic and social values) 393 produced foods (Hempel & Hamm, 2016)

Careful with this conclusion. Urban residents are becoming increasingly interested. Food safety sustainability is now key concerns. Traceability is being demanded. Though this is pressure is yet to become widespread in Africa. You cannot divorce promotion of CBE from sustainability demands. This suggestion is much appreciated and incorporated – we totally drop the explanation of being less concerned but strengthen the increasing reality of urban consumers demanding for safety certified foods. Lines 542 – 544

Line 401-403

Thus, the role of 402 government, next to the private sector’s role for valorisation of waste, seems to be crucial for the 403 establishment of a CBE
This could have been explored as part of the study. Like chemical fertilizer, inorganic fertilizers can also be commercialized by the private sector. This suggestion is appreciated and finally totally dropped in the revised manuscript.

Line 406-407

household food insecurity challenges due to having more persons to feed (Kaza et al. 2018). Hence, 407 resorting to resource conservative and low cost produced foods, such as CBE foods, is logical.
The assumption that CBE foods are cheaper needed to be supported. Indeed, the research should have tried to understand this. This is indeed a critical question that is key to uptake of CBE and we cannot make this assertion without any supporting evidence. We re explain this better with supporting evidence. Lines 574 – 579

Line 419-427

Finally, in DRC, households with severe food insecurity seem to be more likely to eat CBE 420 foods grown using compost, but severely food insecure households in Rwanda and Ethiopia seem 421 less likely to accept eat CBE foods. Interestingly, the effects are not that significant. However, this 422 may be due to some social-cultural values, yet these need longer time to change (Inglehart & Baker, 423 2000). Moreover, in some instances, social stigma has been found to negatively influence food 424 acceptance by food insecure persons/households (Pogash, 2008; Purdam et al. 2015). Therefore, 425 CBE innovations must be aware of potential social dislikes, and strengthen awareness campaigns 426 on safety, and appropriateness of CBE foods. Wrongful consumer perceptions may generate social 427 stigma against CBE foods
It makes no sense to say that the results were not that significant and then go ahead to explain the results and offer potential explanation. Results are not significant means not effect. Any explanation is of no use. Or do you mean the effects are significant but very small. This guidance is well appreciated – we abandon the explanation and explain only significant variables. Lines 547 – 568.

Line 437-440

We recommend that experimental or panel data could be used in the future to support this initial 438 research. That said, we are confident that our results reflect the CBE status quo in the studied city 439 regions and can reliably inform successful implementation and scaling of CBE innovations in those 440 regions
Given that this was an exploratory study , the outhors this should develop a section to explore some hypothesis for other to test in the future. This can be developed from the various conclusion you have made i.e.
i. CBE innovations most likely need to prioritize knowledge and learning components in 363 setting up CBE innovations.
ii. Thus, CBE innovations could target agricultural households for demand for compost and 371 animal feeds
iii. Therefore, 376 CBE innovations could need to prioritize household food security to succeed
iv. hus, CBE 384 innovations should prioritize commercial viability of CBE products to attract male headed 385 (majority) households to agriculture based on CBE practices.
v. Thus, the role of 402 government, next to the private sector’s role for valorisation of waste, seems to be crucial for the 403 establishment of a CBE
vi. Thus, CBE innovations could target households with heads of average old 412 working age, as these could even be more likely to accommodate behavioural changes. With better 413 education, understanding of concepts like CBE becomes possible (Evans et al. 2012) a
vii. Therefore, CBE innovations need to incorporate mechanisms that 418 ensure products’ safety and quality
viii. Therefore, 425 CBE innovations must be aware of potential social dislikes, and strengthen awareness campaigns 426 on safety, and appropriateness of CBE foods. Wrongful consumer perceptions may generate social 427 stigma against CBE foods. This suggestion is much appreciated and incorporated in lines 622 – 640.

Line 446 and line 448-450

We reviewed country policies on CBE practices
Furthermore, policies concerning circular bioeconomy 449 appear to be insufficient, and thus need immediate attention for formulation, clarity, and 450 enforcemen

What was done does not meet the criteria of a review. This was a quick and dirty approach. Need to interview a few actors in policy making and also NGOs and also review grey literature to better understand the policies.	We elaborate that we had originally done expert and key informant interviews in ministries, and municipalities, and project partners, and as well we reviewed grey literature in government reports. Lines 227 – 241.
A study done by Bio innovate on East Africa bioeconomy strategy that included Rwanda and Uganda can give some insights
General insights. Much appreciated for the insights from this study – especially in phrasing what I had done, only that I found this more applicable for a reference: *Gatune, J., Ozor, N. and Oriama, R. (2021), "Modelling bioeconomy futures for Eastern Africa", Foresight, 23 (2): 226-241.* [*https://doi.org/10.1108/FS-03-2020-003*2](https://doi.org/10.1108/FS-03-2020-0032). Lines 112, 127, and 530

It would also have been good to reflect on what your results mean in terms of market for CBEs and implication for value chain development. If demand is there in urban areas should we target a certain segment. For example organic foods tend to target higher income. Much appreciated suggestion and incorporated in manuscript. Lines 653 – 654.

Also given the need to have waste re-used at rural level how should value chain be re-organized to help lower the cost of taking waste back to rural areas?. This is much appreciated and incorporated in lines 659 – 661.
