# Supplementary material for: Circular bioeconomy in African food systems: What is the status quo? Insights from Rwanda, DRC, and Ethiopia
Source: PLoS One. 2022 Oct 20;17(10):e0276319. doi: 10.1371/journal.pone.0276319 (PMC9584527; doi:10.1371/journal.pone.0276319)
Supplement: Attachment — Submitted filename: Reviewer Comment.docx [file pone.0276319.s005.docx]

It is an interesting study but the authors lack certain adjustments to make in the text so that the work is comprehensive to the readers and so that the article is accepted.

Abstract

I think it is necessary to list in the summary all the statistical instruments used throughout the research and not only talk about logistic regression. It's like it was the only statistical method used.

Introduction

What kind of organic residue are the authors referring to? It's a little confusing in the text. Should these organic residues they are talking about be used only for cassava, banana and coffee crops? This part is not very clear in the introduction and other parts of the text.

I suggest that the authors create after the introduction, a section for the circular economy, the bioeconomy and then for the circular bioeconomy before the work methodology section.

Methodology

In the methodology, the authors have not demonstrated through statistical calculations how they obtained the samples of interviewees in each country. They did not specify whether in each country the sample was probability or not. This may confuse the reader. It must clearly state in the method the categories of people who responded to the questionnaires. Also, tell each country how long it took to collect the data.

Why did you opt for data analysis using descriptive analysis and logistic regression? What is the importance of using this statistical tool?

Are the people who collected the data specialists, students, and professors? It is important to say so in the methodology.

Result

The percentages of the different respondents in each country quoted above in Table 4 do not appear in the latter. The authors need to review this part.

Figure 1 mentioned by the authors does not appear in the text. I suggest a compilation of tables 5 to 10, presenting only the main results.

Analysis and discussions

I suggest that the authors discuss the different categories, CBE practices; awareness, knowledge, and support for CBE practices; consumers’ opinions, explored in their tabular results, adding the different observations for each country.

As an example, authors can consult the article by Mourad (2016).

Mourad, Marie (2016). *Recycling, recovering and preventing “food waste”: competing solutions for food systems sustainability in the United States and France. Journal of Cleaner Production, (), S0959652616301536–.* doi:10.1016/j.jclepro.2016.03.084

Conclusions

Acceptable.
